# Colon organoid formation and cryptogenesis are stimulated by growth factors secreted from myofibroblasts

**DOI:** 10.1371/journal.pone.0199412

**Published:** 2018-06-21

**Authors:** Hon Yan Kelvin Yip, Chin Wee Tan, Yumiko Hirokawa, Antony Wilks Burgess

**Affiliations:** 1 Structural Biology, Walter and Eliza Hall Institute of Medical Research, Parkville, Victoria, Australia; 2 Department of Medical Biology, University of Melbourne, Parkville, Victoria, Australia; 3 Department of Surgery, University of Melbourne, Royal Melbourne Hospital, Parkville, Victoria, Australia; University of North Carolina at Chapel Hill School of Medicine, UNITED STATES

## Abstract

Although small intestinal epithelial stem cells form crypts when using intestinal culture conditions, colon stem cells usually form colonospheres. Colon mesenchymal cell feeder layers can stimulate colon crypts to form organoids and produce crypts. We have investigated whether conditioned medium from colon mesenchymal cells can also stimulate colonosphere and organoid cryptogenesis. We prepared conditioned medium (CM) from WEHI-YH2 cells (mouse colon myofibroblasts); the CM stimulated both colonosphere formation and organoid cryptogenesis *in vitro*. The colon organoid-stimulating factors in WEHI-YH2 CM are inactivated by heating and trypsin digestion and proteins can be concentrated by ultrafiltration. Both the colonosphere- and organoid cryptogenesis- stimulatory effects of the CM are independent of canonical Wnt and Notch signaling. In contrast, bone morphogenetic protein 4 (BMP4) abolishes colonosphere formation and organoid cryptogenesis. The Transforming Growth Factor beta (TGFβ) Type I receptor kinase inhibitor (A83-01) stimulates colonosphere formation, whereas the Epidermal Growth Factor receptor (EGFR) kinase inhibitor (AG1478) reduces the formation of colonospheres, but in the presence of EGF, a “just-right” concentration of AG1478 increases colon organoid cryptogenesis.

## Introduction

Colon epithelium is composed of a continuous sheet of epithelial cells folded into glandular crypts. The maintenance of crypt cells is regulated by stem cells at the bottom of crypts [1]. The stem cells not only produce new cells within a crypt, they are also responsible for generating new crypts. Leucine-rich repeat-containing G-protein-coupled receptor 5 (LGR5), has been identified as an epithelial stem cell marker in the small intestine and the colon [2]. At the bottom of the crypt LGR5^+^ stem cells are self-renewing [2] and are capable of producing progenitor cells which in turn form the mature crypt cells (e.g. colonocytes, goblet cells, enteroendocrine cells and tuft cells) of the intestinal epithelium. Other stem cells like Leucine Rich Repeats And Immunoglobulin Like Domains 1 (Lrig1)+ cells[3] and Doublecortin Like Kinase 1 (Dclk1)+ cells [4] were also identified. However, it is not yet clear which type(s) of intestinal stem cells can form new crypts.

During organogenesis or upon intestinal damage, there is increase in circumference of the intestine [5] (i.e. the surface area of the intestine increases and new crypts are produced). Similarly, during repair of the intestinal epithelium [6] new crypts are produced by crypt budding (previously referred to as crypt fission)[7]. Crypt buds form in the stem cell compartment and project out from the crypt axis [5,8,9]. The bud then elongates to form a full-sized daughter crypt with functional mature cells. This process is associated with proliferation of stem/progenitor cells and differentiation of progenitor cells into mature cells. Increased crypt budding has been described as the mechanism driving the growth of human colorectal hyperplastic polyps [10], but it also correlates with adenoma formation [11]. There is a higher incidence of asymmetric crypt budding in both the small intestinal and colonic epithelium of Apc^min^ mice [12] and in the intestinal epithelium of human familial adenomatous polyposis patients [12]. The mechanisms of how niche factors regulate crypt initiation, crypt survival or intra-crypt cell production in normal, adenomatous or cancerous colonic mucosa is still unclear.

Tissue re-sorting experiments demonstrated that the developing intestinal endoderm and mesenchyme influence one another during tissue development [13]. Extensive studies using transgenic mouse models show that mesenchymal factors e.g. epimorphin [14], bone morphogenetic proteins (BMPs) [15,16], Wnts [17–19], EGF [20], TGFβ [21] and Hedgehog [17] can influence villus-crypt formation in the intestine. Most of these studies have focused on the small intestine rather than the colon, there is some indication that the colonic mesenchyme can influence the proliferation and differentiation of colonic epithelium during development [22]. Due to difficulties associated with real-time *in vivo* imaging of the intestine in animal models, experiments to study the effects of microenvironmental cells on colon crypt budding have been difficult. Recently, the development of small intestine [23] and colon [24,25] organoid cultures has allowed the direct study of both intra-crypt cell production and crypt budding in organoids developed from both small intestinal and colon stem cells.

3D organoid cultures were first developed for murine small intestine stem cells [23] but similar techniques are now used to grow murine colon organoids [24,25] and human colon tissue and biopsy [26] *in vitro*. Different terminologies are used to describe the results from the various types of cultures: the terms enterospheres (spheroids) and enteroids (spheroids with crypt-like projections) are used to describe the structures formed from *small intestinal* crypts/cells in Matrigel-embedded cultures [27]; the terms colonospheres (organoids without projections) and colonoids (defined as organoids with crypt-like projections) have been used to describe similar structures formed from *colon crypts*/cells [27,28]. We have previously reported results from our co-culture system using isolated colon crypts and the colon myofibroblast cell line WEHI-YH2 [29] where the WEHI-YH2 cells stimulated the formation of colonospheres and colonoids [29]. This co-culture system has a higher efficiency for the formation of colonoids (i.e. organoids with crypt-like projections) [24,30,31].

We have noticed that when colon crypts are cultured in [29] or on top of Matrigel[32], there is an initial transformation to small colonospheres (i.e. in the first day of culture), but as the cultures progress the colonospheres develop into oblate ellipsoids from which crypt budding is initiated. In this study, we report the effects of proteins agonists and inhibitors of selected signaling pathways and the conditioned medium from WEHI-YH2 cells (a colon myofibroblast cell line [29]) on colonosphere formation and cryptogenesis *in vitro*.

## Results

**Fig 1:**
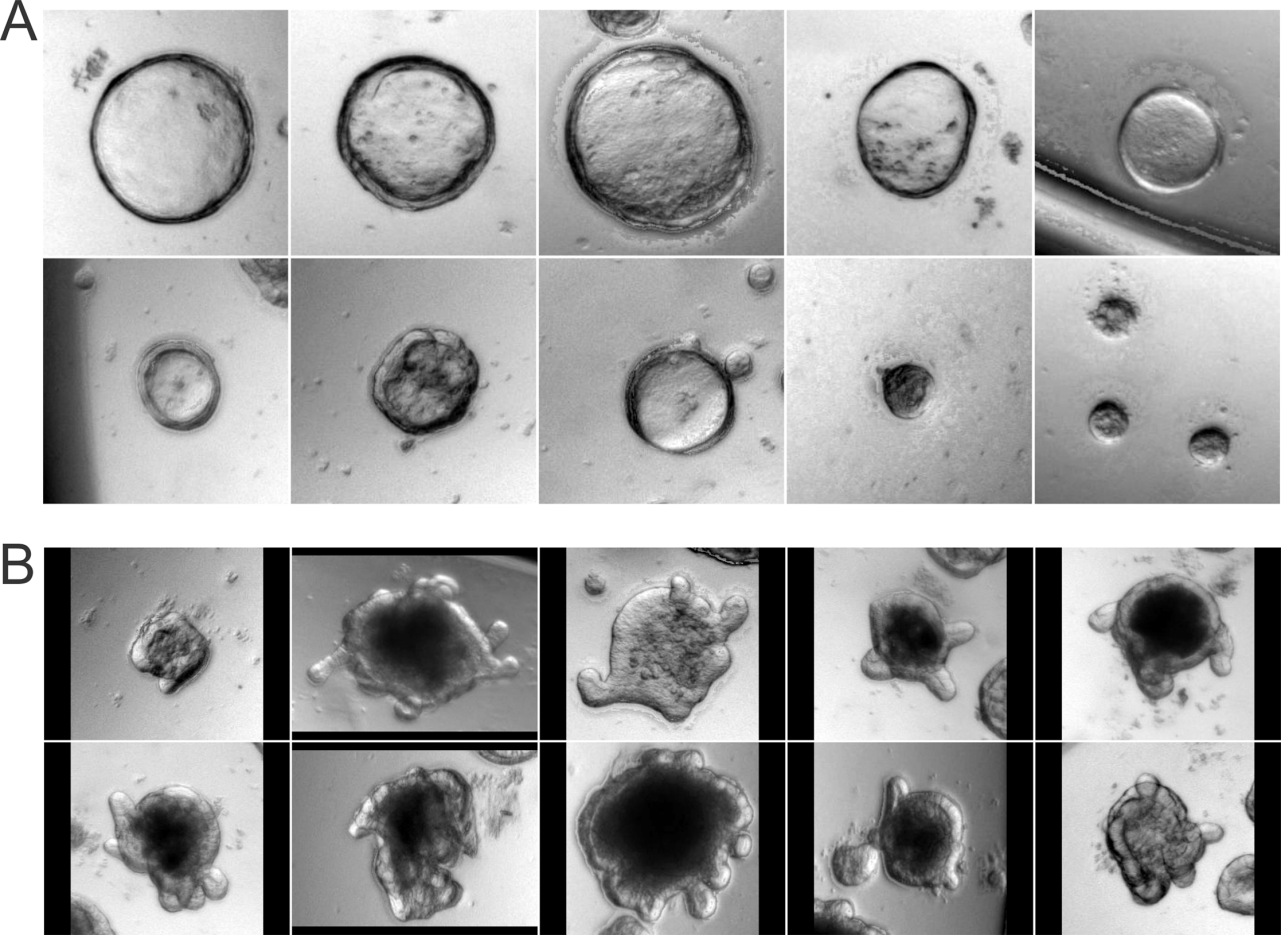
Colonospheres and organoids with crypts (budding organoids) at day 6 of culture. Colon crypts were isolated from mouse and cultured embedded with Matrigel in growth factors containing DMEM/F12 media. Two distinct morphologies of cultures can be identified, namely: (A) Colonospheres, which are spherical or cystic in shape without crypt-like projections. (B) Colonoids, which are oblate ellipsoids shaped structures with crypt-like buds extending outwards into the Matrigel.

Mouse colon myofibroblasts (WEHI-YH2 cells) feeder layers stimulate the formation and size of colonospheres and colon organoids with budding crypts [29]. The stimulation of crypt formation does not require cell-cell contact, as the WEHI-YH2 cells can be separated from the crypt/Matrigel cultures by a permeable membrane [24]. In this study, we investigate the activity of the conditioned medium produced by the WEHI-YH2 cells (termed YH2CM) on colonosphere and colon crypt formation in vitro (Fig 1).

### YH2CM stimulates colonoid formation from FACS-sorted cells enriched for putative intestinal stem cell markers

**Fig 2:**
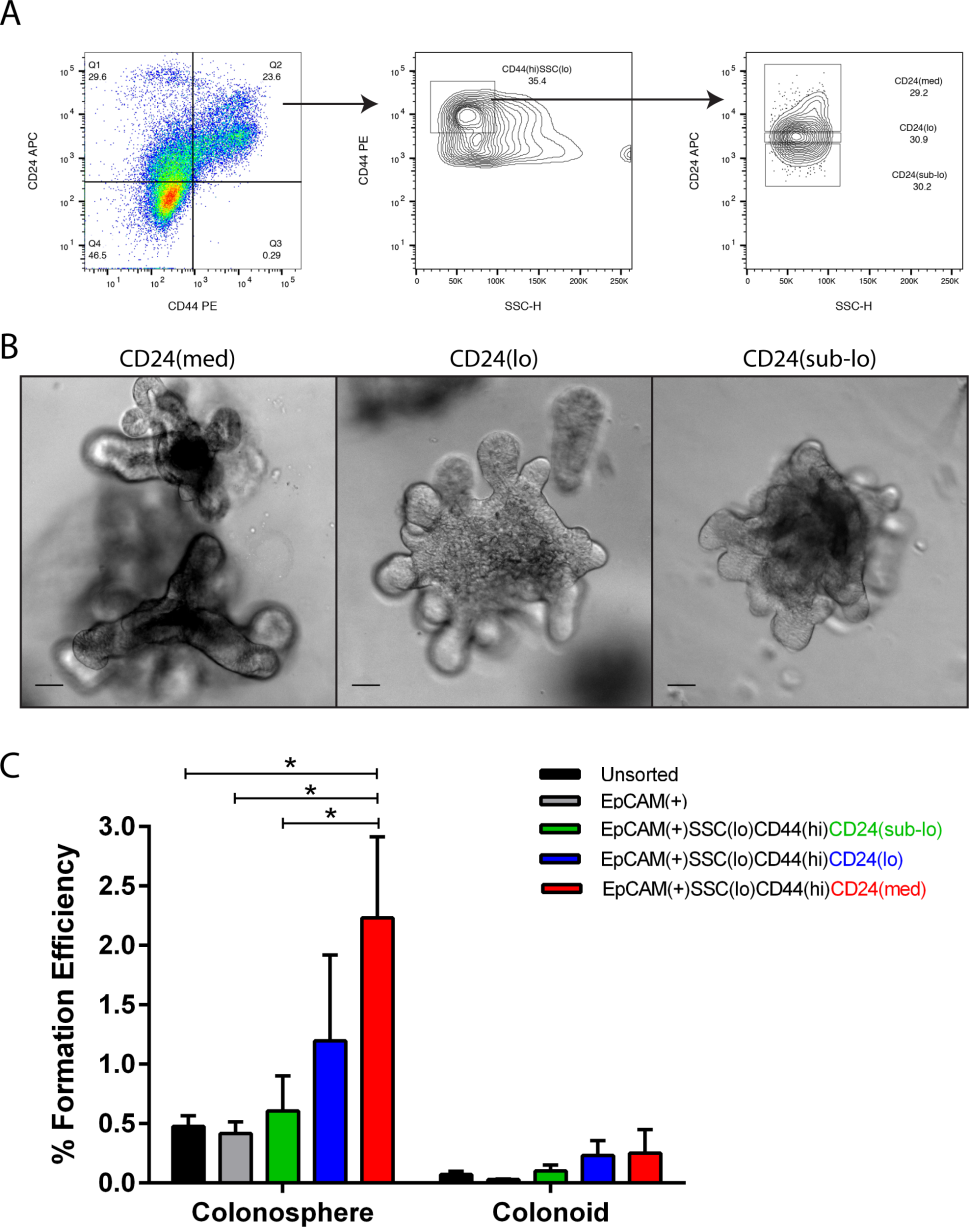
WEHI-YH2 feeder layer co-culture stimulates stem cells in colon crypt cultures. Epithelial cells were purified using EpCAM-PE/Cy7, CD44-PE and CD24-APC antibodies. (A) The CD44^+^CD24^+^ cells in quadrant 2 in the bivariate plot were gated. A further gate on SSC^lo^CD44^hi^ subset followed by CD24^sub-lo/lo/med^ subsets were used to purify colon epithelial cells into 3 further subsets: EpCAM^+^SSC^lo^CD44^hi^CD24^sub-lo^, EpCAM^+^SSC^lo^CD44^hi^CD24^lo^ and EpCAM^+^SSC^lo^CD44^hi^CD24^med^. (B) Representative images of colonoids formed after the 3 subsets of colon cells were embedded in Matrigel and co-cultured with a monolayer of WEHI-YH2 cells for 8 days. Scale bar = 50 μm. (C) The formation efficiency (%) of colonospheres and colonoids in day 8 culture. Error bars: SEM n≥3, 1-way ANOVA, *p<0.05.

We have shown previously that both YH2CM and WEHI-YH2 feeder cells stimulates colon crypt formation in colon crypt cultures with new crypts formed from colonospheres[29]. This is indicative that these colonic crypts cultured contain cells capable of generating all the different cell types in a colon crypt, i.e. including the stem cells. To show that YH2CM contains factors that stimulate/maintain colon crypt stem cells in our *in vitro* cultures, colon stem cells were isolated and enriched by flow cytometry using a EpCAM^+^CD44^hi^CD24^med^ signature previously reported [33,34] (Fig 2A). The enriched population of cells were co-cultured with WEHI-YH2 cells in Matrigel 3D cultures and imaged daily for 6 days. The EpCAM^+^SSC^lo^CD44^hi^CD24^med^ cell population are able to forms colonoids similar to those derived from colon crypts (Fig 2B and 2C). This shows that WEHI-YH2 cells produce factors which promote colonoid formation from colon stem cells.

### Ultrafiltration concentrated conditioned medium from WEHI-YH2 cells stimulates colonosphere formation and cryptogenesis as effectively as WEHI-YH2 feeder cells

**Fig 3:**
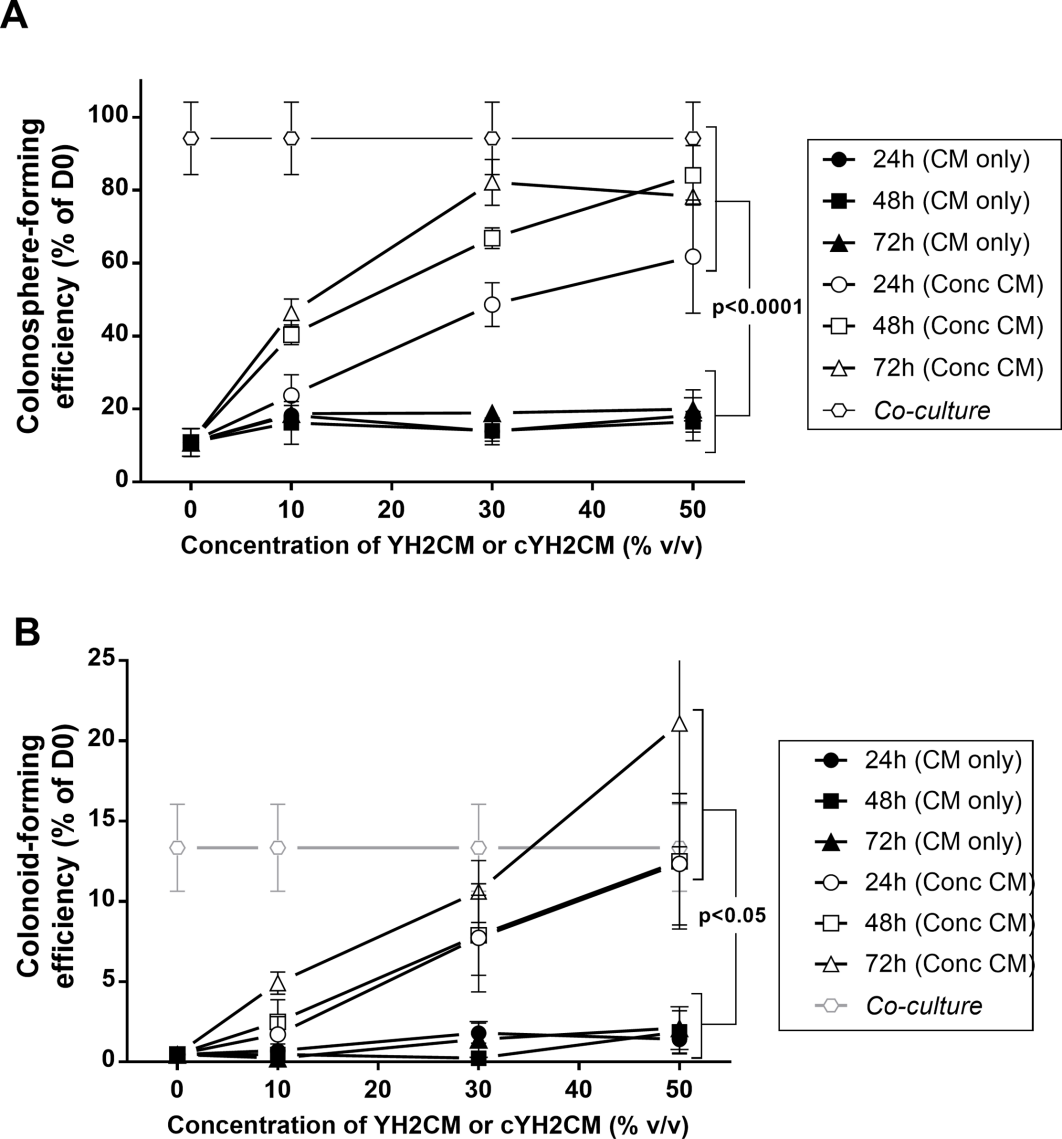
Concentrated medium collected from WEHI-YH2 cells (cYH2CM) stimulates colon crypt formation. Conditioned Medium (CM) from WEHI-YH2 cells (YH2CM) was collected from confluent cultures incubated for different times (24, 48 and 72 hours) in DMEM/F12 with the N2 and B27 supplements. The CM was concentrated 10-fold using a Centriprep YM3 filter (with a 3kDa molecular weight cut-off) (cYH2CM). Different amounts of YH2CM and cYH2CM were used to grow colon crypts in culture and the results are compared with the WEHI-YH2 colon crypt co-cultures. Images of the cultures were taken on Day 0 and Day 6, processed and scored for either colonospheres or organoids with crypts: (A) colonosphere- and (B) colon crypt–forming efficiency on Day 6 (the number of colonospheres or colonoids on Day 6 divided by the number of crypts plated on Day 0). The dotted lines show the colonosphere and colon colonoid formation efficiency in co-cultures with WEHI-YH2 cells. Error bars: SEM, n = 4 from 2 independent experiments of duplicates, 2-way ANOVA multiple comparisons Tukey’s test.

Colon crypts were grown in 3D Matrigel cultures (see materials and methods) using either un-concentrated YH2CM or concentrated YH2CM (up to 50% v/v of the medium) where they form colonospheres or colonoids. The formation of colonospheres and cryptogenesis stimulated by WEHI-YH2 feeder layer cultures or YH2CM are compared (Figs 3A and 2B). After 6 days in the Matrigel cultures, in the absence of feeder layers, mouse colon crypts produce colonospheres with an efficiency of <11% (i.e. for every crypt cultured with Matrigel 11% formed a colonosphere, Fig 3A) but <1% of the crypts produced an organoid with budding crypt(s) (Fig 3B). In the presence of WEHI-YH2 cell feeder layer, the colonosphere formation efficiency at day 6 increased to >90% and 13% of the organoids had budding crypts. When used as 50% v/v as unconcentrated culture medium, YH2CM (prepared for 24, 48 or 72 hours) only stimulates a small number of colonospheres and budding crypts at day 6 (Fig 3A and 3B). In contrast, after a 10-fold concentration of the YH2CM using a Centriprep^®^ YM3 ultrafiltration membrane with a 3 kDa molecular weight cut-off (termed **cYH2CM**), colonosphere formation and cryptogenesis were both stimulated 7- and 8-fold respectively (compared to controls) (Fig 3A and 3B). cYH2CM, harvested after 24, 48 or 72 hours, all stimulated colonosphere and crypt formation, with the 72hr CM significantly more effective than the 24h or 48h CM in stimulating cryptogenesis. Even when used at 10% (v/v), the cYH2CM increased colonosphere and colonoid formation while at 50% (v/v), the cYH2CM produced more colonoids than that produced in the feeder layers (i.e. co-cultures) cultures. As such, concentrated WEHI-YH2 conditioned medium (cYH2CM) is able to stimulate crypt formation (cryptogenesis) and is as effective as cultures that uses WEHI-YH2 as feeder cells.

It should be noted that these cultures contained all the reported factor(s) required for the formation of small intestinal enterospheres and enteroids. For small intestinal crypt cultures, the organoid forming efficiency was close to 100% using this cocktail of factors [24], without using either the WEHI-YH2 feeder layers or the cYH2CM. However, colon crypts only formed colonospheres and crypts efficiently when they were stimulated by either WEHI-YH2 feeder layers or the cYH2CM.

For characterizing the factors present in YH2CM, it was preferable to reduce the protein complexity of the YH2CM. In the initial experiments, as described above (Fig 3A and 3B), media supplements (N2 and B27, which contain superoxide dismutase, insulin, bovine serum albumin (BSA), transferrin and catalase (Life Technologies #17502048 and #17504)) had been included in the medium used to prepare the YH2CM. For all subsequent experiments the YH2CM was prepared in serum-free, supplement-free DMEM/F12. The removal of these supplements (N2 and B27) had no effect on the ability of cYH2CM to stimulate colonosphere or colonoids (see S2 Fig).

**Fig 4:**
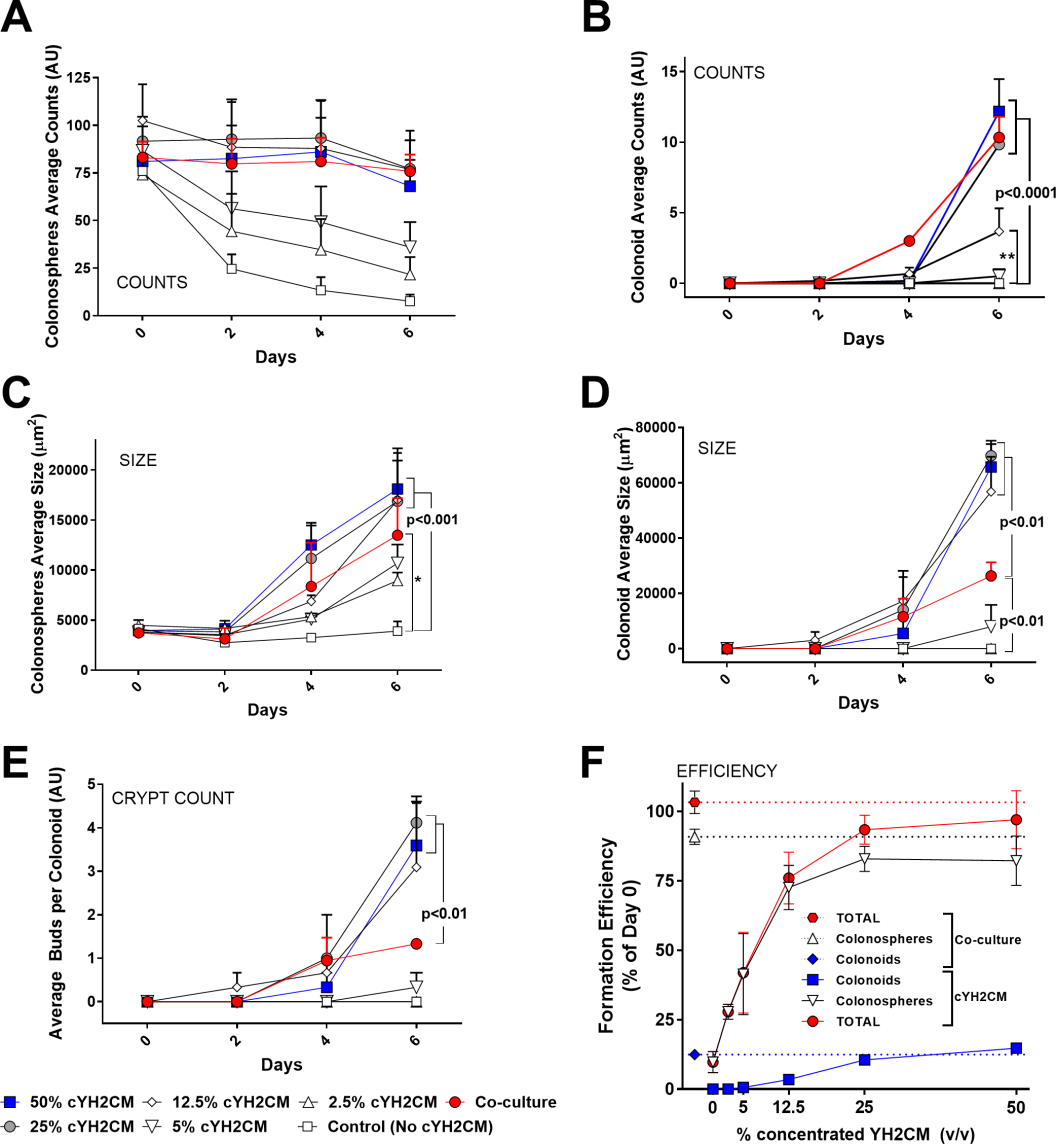
cWEHI-YH2 CM stimulates colon crypt formation maximally. Different proportions (2.5% to 50%, v/v) of ten-fold concentrated YH2CM conditioned media (cYH2CM, using the Vivaflow 50R) was used to grow colon crypts in culture. Corresponding co-cultures with WEHI-YH2 cells and without cYH2CM were used as positive and negative control respectively. Whole well image stacks of the cultures were acquired on days 0, 2, 4 and 6. Images were quantitated for average counts, sizes, crypts per organoid as well as the efficiency for formation of Colonosphere (A and C) and colonoid (B, D-F). For colonosphere formation, 12.5% of cYH2CM is sufficient to match the effect of YH2-CM co-culture in maintaining spheroid counts (A) and promoting increase in spheroid size (C). For forming colonoids, 25% cYH2CM was needed to match the effect of the WEHI-YH2 feeder layers in terms of counts (B) and proliferation (D). (E) When used at ≥12.5% (v/v) the cYH2CM improves the average crypt budding rate per colonoid significantly (compared to the co-cultures). (F) The colonosphere and colonoid formation efficiencies at Day 6 (compared to Day 0) using ≥25% cYH2CM (v/v) is comparable to the WEHI-YH2 co-cultures. Error bars: SEM, n = 3 experiments of duplicates, 2-way ANOVA multiple comparisons (A, B) Dunnett test, (C,D,E) Tukey’s test.

The supplement free cYH2CM was titrated from 50% to 2.5%, v/v into the Matrigel cultures of colon crypts and the cultures were imaged on days 0, 2, 4 and 6. The number of colonospheres/colonoids, their areas and the number of crypts per colonoid were measured and compared to those of the co-cultures (Fig 4 and S3 Fig). When the cYH2CM is used at <12.5%, v/v, the colonosphere number decreases from day 0 to day 6 (Fig 4A). At the higher cYH2CM concentrations (12.5%, 25% and 50%, v/v), colonosphere counts remain stable and comparable to the numbers formed in the co-cultures (Fig 4A, dotted lines). Colon crypt formation typically begins by day 4 and increases in number by day 6 (Fig 4B). The number of colon organoids with crypts formed when using 25% v/v or 50% v/v cYH2CM was comparable to the number stimulated by the WEHI-YH2 co-cultures (Fig 4B). Without cYH2CM (control) or WEHI-YH2 feeder layers (co-culture), none of the initial crypts produced organoids with budding crypts by day 6 (Fig 4B). Without cYH2CM, the colonospheres did not increase in size (Fig 4C). Using cYH2CM at concentrations ≥12.5% v/v led to significantly larger colonospheres and colonoids at day 6 (Fig 4C). Interestingly, the cYH2CM (≥12.5% v/v) stimulated significantly more crypts than the WEHI-YH2 feeder layers (Fig 4E). Fig 4F shows a dose-response curve of the formation efficiency of colonospheres and colonoids at different cYH2CM concentrations on day 6. The maximum formation of colonospheres and colonoids was achieved with 25% v/v cYH2CM (Fig 4F).

### Biochemical characterization of the YH2CM

**Fig 5:**
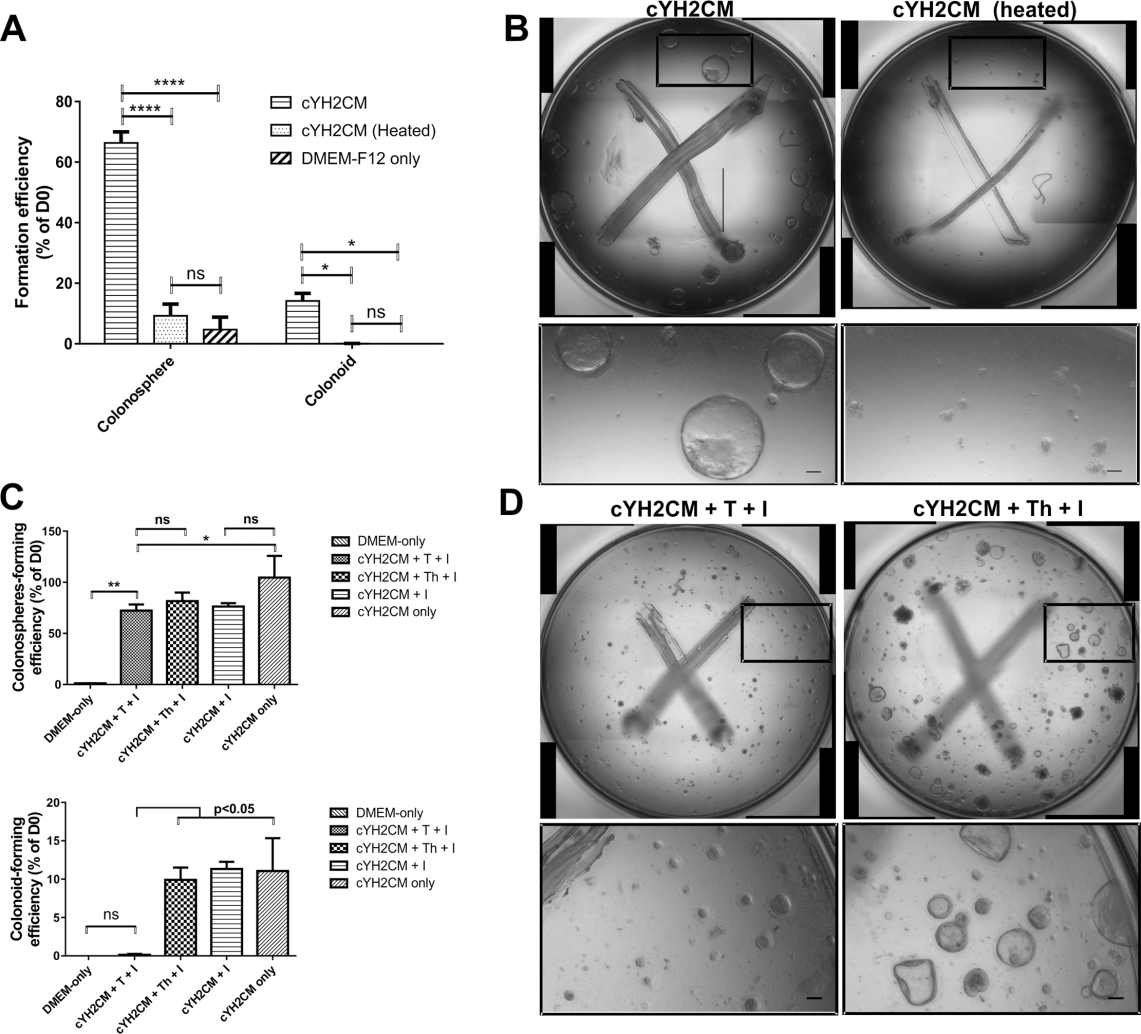
Factors simulating colon crypt cultures appear to be proteins. To determine the biochemical characteristics of the factors that simulate colon crypt formation *in vitro*, thermal or enzyme treatments were applied to concentrated YH2CM-DMF conditioned media (cYH2CM). The treated cYH2CM were used to stimulate colon crypts in culture at 50% v/v and the results compared with that of the untreated cYH2CM. Whole well image stacks of the cultures were acquired on days 0 and 6. Images were quantitated and the formation efficiency of colonospheres and colonoids (% of Day 0) was calculated. (A) Heat treatment of cYH2CM at 68°C for 1 hour (denatured) obliterates the ability to grow colon crypt cultures. (B) Representative images showing the colonospheres formed in the presence of in cYH2CM, compared to no colonospheres in the cultures treated with the heat denatured cYH2CM. (C) cYH2CM was pre-treated with 100 μg/ml of trypsin (*T*) or denatured trypsin (*Th*, heated at 95°C for 10 minutes) at 37°C for 6 hours before deactivating the trypsin with 400 μg/ml of trypsin inhibitor (*I*). Trypsin treatment completely obliterates colonoid forming capability and reduces the colonosphere forming activity of cYH2CM. (D) Representative images of colon crypt cultures showing the differences between trypsin-treated cYH2CM cultures and cultures treated with inactivated trypsin-treated cYH2CM. Scale/Error bars and analysis: (A) ±SEM n = 4, Tukey's multiple comparisons test. (C) Scale bar: 50 μm, ±SEM n = 3, 1-way ANOVA multiple comparisons, Uncorrected Fisher's LSD test.

It is necessary to determine the nature of the factor(s) in the YH2CM before attempting to purify or identify the molecules responsible for either stimulating the growth of the colonospheres or for stimulating the colon organoids to form crypts. To characterize stability of the factors in the YH2CM that promote colonoid and colonosphere formation (collectively termed colonoid-stimulating factors, CoSFs), the cYH2CM was heated at 68°C for an hour [35]—to denature the proteins in the conditioned medium. Heating the cYH2CM significantly destroyed the CoSF activity (Fig 5A). The few colonospheres remaining in the cultures stimulated with the heated cYH2CM were much smaller than those found in untreated cYH2CM (Fig 5B). This loss of colonosphere/crypt-stimulating activity suggests the CoSFs in cYH2CM are likely to be proteins. This is consistent with the observation that trypsin (but not heat denatured trypsin) also reduced the CoSF (Fig 5C and 5D). Although trypsin digestion of cYH2CM only reduced the efficiency of colonosphere formation by 32% (Fig 5C), the remaining colonospheres were much smaller with the trypsin-treated CoSF (Fig 5D). Trypsin digestion abolished the colonoid forming activity of the CoSF completely (Fig 5C, lower panel). Our results indicate that the CoSFs are likely to be proteins.

### Effects of YH2CM on Wnt and Notch signaling

**Fig 6:**
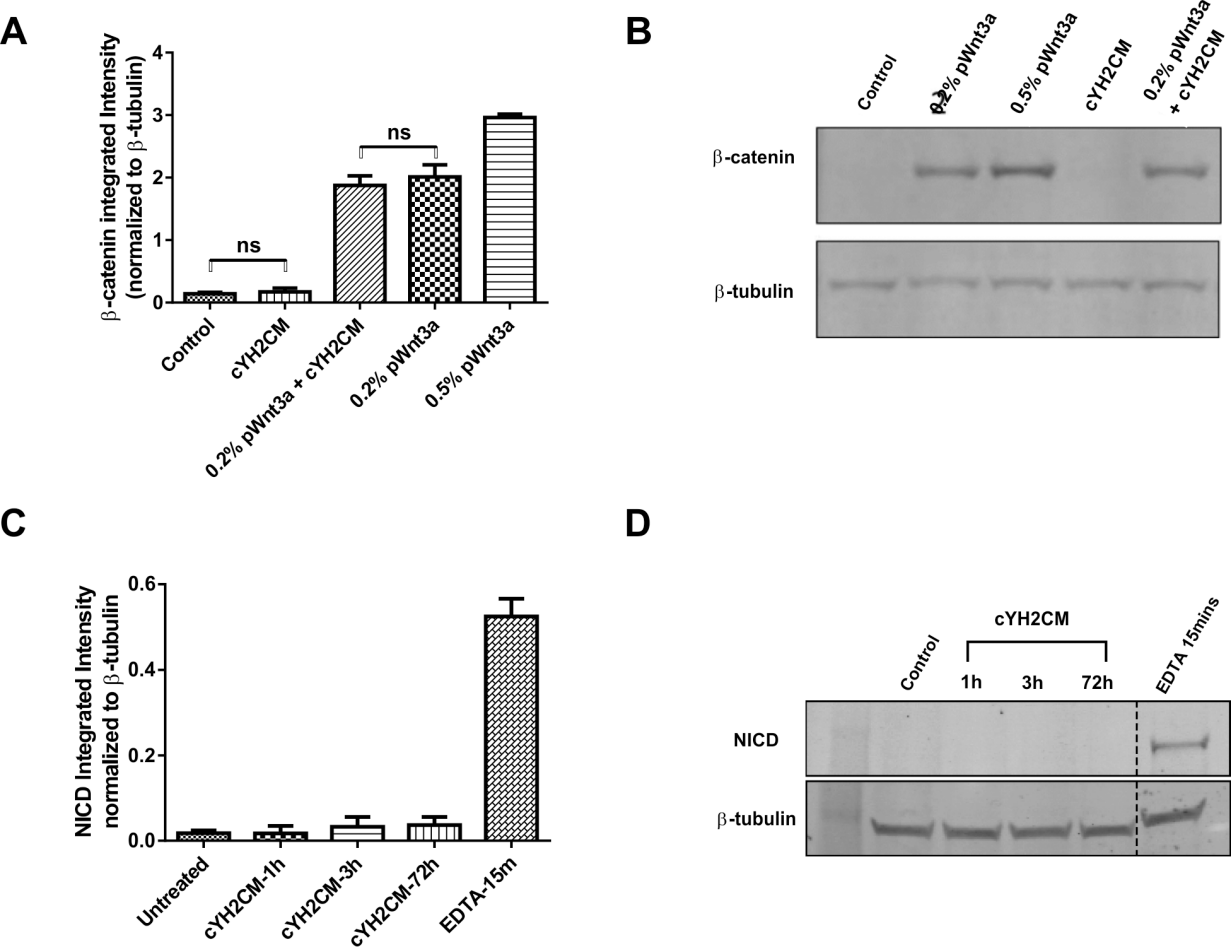
The cYH2CM does not stimulate canonical Wnt and Notch signaling pathways. The ability of the YH2CM to stimulate the Wnt or Notch signaling pathway was tested in cell lines by its ability to stabilize the β-catenin protein (for Wnt pathway) or Notch Intra-Cellular Domain protein (NICD, for Notch pathway). (A) L-cells were incubated with partially purified Wnt3a (0.2% and 0.5% v/v) or cYH2CM (10% and 50% v/v) for 4 hours before Western blotting for β-catenin and β-tubulin. The normalized β-catenin intensities were plotted indicating that cYH2CM does not stabilize β-catenin. (B) Western blot detecting β-catenin using mouse anti-human/mouse β-catenin monoclonal antibody (Clone 14/ β-catenin, BD Transduction Laboratories #610153) to probe the L-cell cell lysate. (C) HCT116 cells were incubated either with 50% (v/v) of DMEM (control) or cYH2CM for 1h, 3h and 72h. For EDTA treatment, the cells were incubated with EDTA/PBS (10 mM) for 15 min. Western blotting for NICD and β-tubulin was conducted on the cell lysate with the normalized NICD intensities plotted. cYH2CM does not stimulate NICD protein in β-catenin. (D) Western blot detecting NICD using rabbit anti-mouse cleaved Notch 1 monoclonal antibody (Clone D3B8, Cell Signaling #4147) to probe the HCT116 cell lysate. Error bars: SEM, n = 3, 2-way ANOVA Tukey’s test.

In intestinal biology, the stem cells are regulated by extracellular proteins [18,36]. The canonical Wnt signaling and Notch signaling have been implicated in the self-renewal, survival and proliferation of stem cells in the crypts [37–39]. The colonosphere/crypt cultures all contain optimal concentrations of Wnt, R-spondin and EGF, so the CoSFs are unlikely to influence colonosphere or colonoid formation through these signaling pathways. Indeed, WEHI-YH2 cells do not express Wnt3A or R-spondin 2 proteins [29] (S4 Fig). However, these two factors are not the only activators of canonical Wnt signaling pathway, we measured the activity of cYH2CM on canonical Wnt signaling by analyzing the levels of β-catenin in L-cells after 4 hours of cYH2CM treatment (**Fig 6**) [40]. Partially purified Wnt3a conditioned medium (pWnt3a) stimulated the accumulation of β-catenin, but cYH2CM, even at 50%, v/v, failed to stimulate the accumulation of β-catenin directly, nor did it synergize with the effects of Wnt3A CM (**Fig 6A and 6B**). cYH2CM does not stimulate the canonical Wnt signaling.

The effect of cYH2CM on Notch signaling pathway was measured by assessing the level of Notch Intracellular Domain (NICD) in HCT116 cells after incubation with cYH2CM (50%, v/v) for 1, 3 and 72 hours. cYH2CM did not stimulate NICD production (**Fig 6C and 6D**).

### Effects of BMP, TGFβ and EGF signaling on colonoid formation in the presence of cYH2CM

Several other signaling pathways have been implicated in the regulation of cell production in intestinal crypts, namely Bone Morphogenetic Protein (BMP), Transforming growth factor-β (TGFβ) and Epidermal growth factor (EGF) signaling [41–43]. Consequently, we have investigated whether any of component of the CoSF activity might be associated with these protein families.

BMP antagonist gremlin 1, gremlin 2 and chordin-like 1 proteins are known to be expressed in the human colon mesenchyme [44]. When noggin (a BMP antagonist, which has been reported to be important for stem cell survival in intestinal crypts [41]) is over-expressed in mouse, *de novo* crypts form in the villi of the small intestine [16]. By manipulating the BMP and TGFβ signaling pathway, Reynolds *et al*. (2014) successfully grew human colon crypts in Matrigel cultures [45]. However, the effects of BMP or TGFβ signaling on colon crypt formation (i.e. colonoid formation) remain unclear.

**Fig 7:**
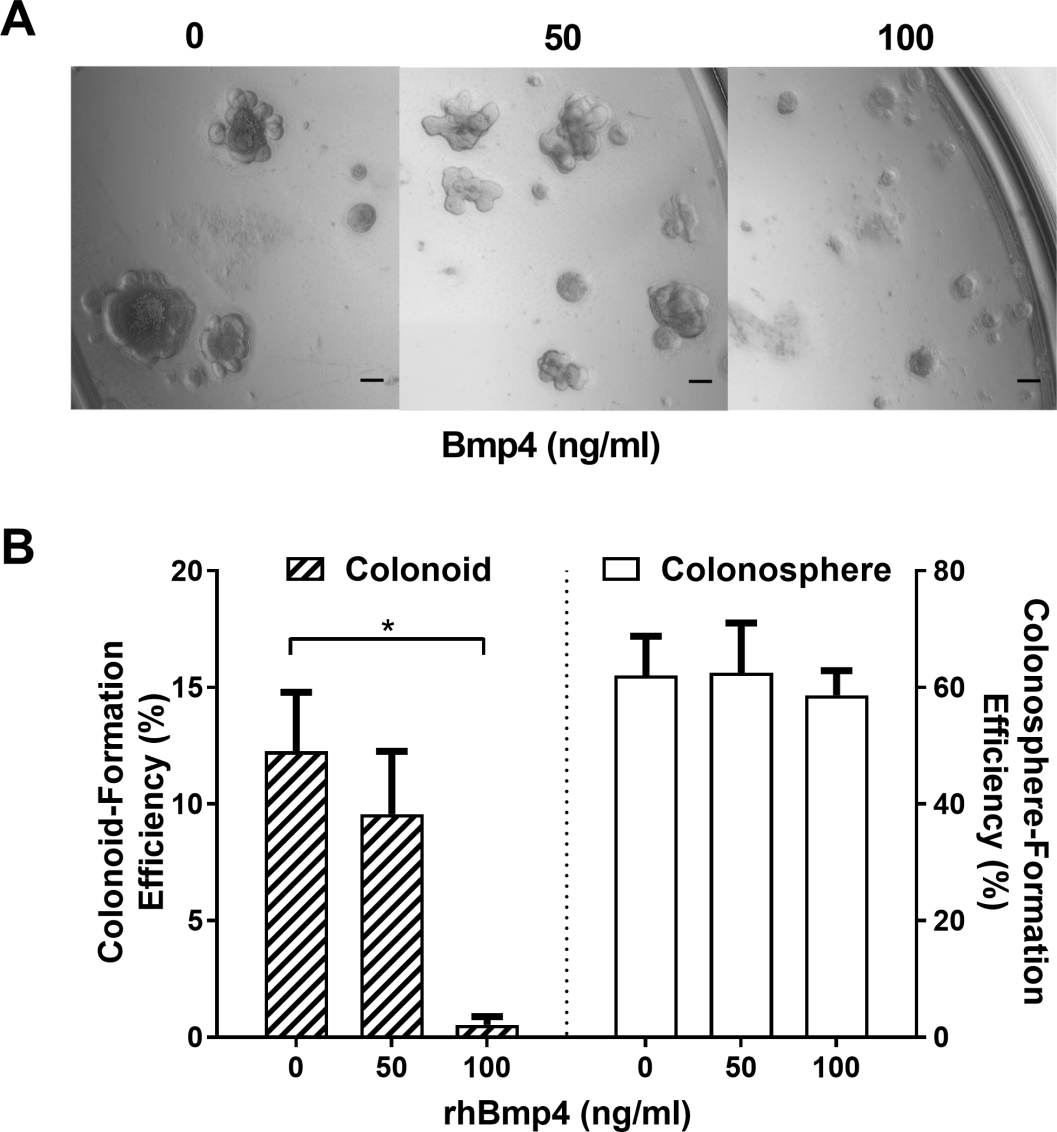
Bone morphogenetic protein 4 (Bmp4) inhibits *colonoid* formation. Recombinant human Bmp4 (50 and 100 ng/ml) and noggin (100 ng/ml) was added to the colon crypt cultures on Day 0, 2 and 4 and assayed in duplicates. (A) Representative bright field *Extended Depth of Field* (EDF) images of the cultures on Day 6. (B) The numbers of colonospheres and colonoids were scored on Day 6 and the colony formation efficiencies were determined and tabulated. Error bars and analysis: Mean ± SEM, * p< 0.05, n = 3, scale bar = 100 μm, 2-way ANOVA multiple comparisons, Tukey’s test.

Noggin is routinely added to our colon crypt Matrigel cultures [23,24]. To investigate the effect of BMP with cYH2CM on colon crypt development, different concentrations of recombinant human BMP4 (rhBMP4) were titrated with 100ng/ml of recombinant noggin in the presence of 50%, v/v of cYH2CM on days 0 and every other day thereafter. The cultures were imaged and formation efficiency of colonospheres and colonoids were scored on day 6 (**Fig 7A–7C**). Colonosphere formation efficiency was not affected by rhBMP4, but crypt formation was significantly reduced at 100 ng/ml of rhBMP4 (**Fig 7C**). The 100ng/ml noggin was sufficient to reduce inhibition of 50ng/ml rhBMP4, higher concentrations of rhBMP4 were able to inhibit colonoid but not colonosphere formation [41] (**Fig 7C**). It appears that BMP signaling [41] does not regulate colonosphere formation but inhibits colon crypt formation.

**Fig 8:**
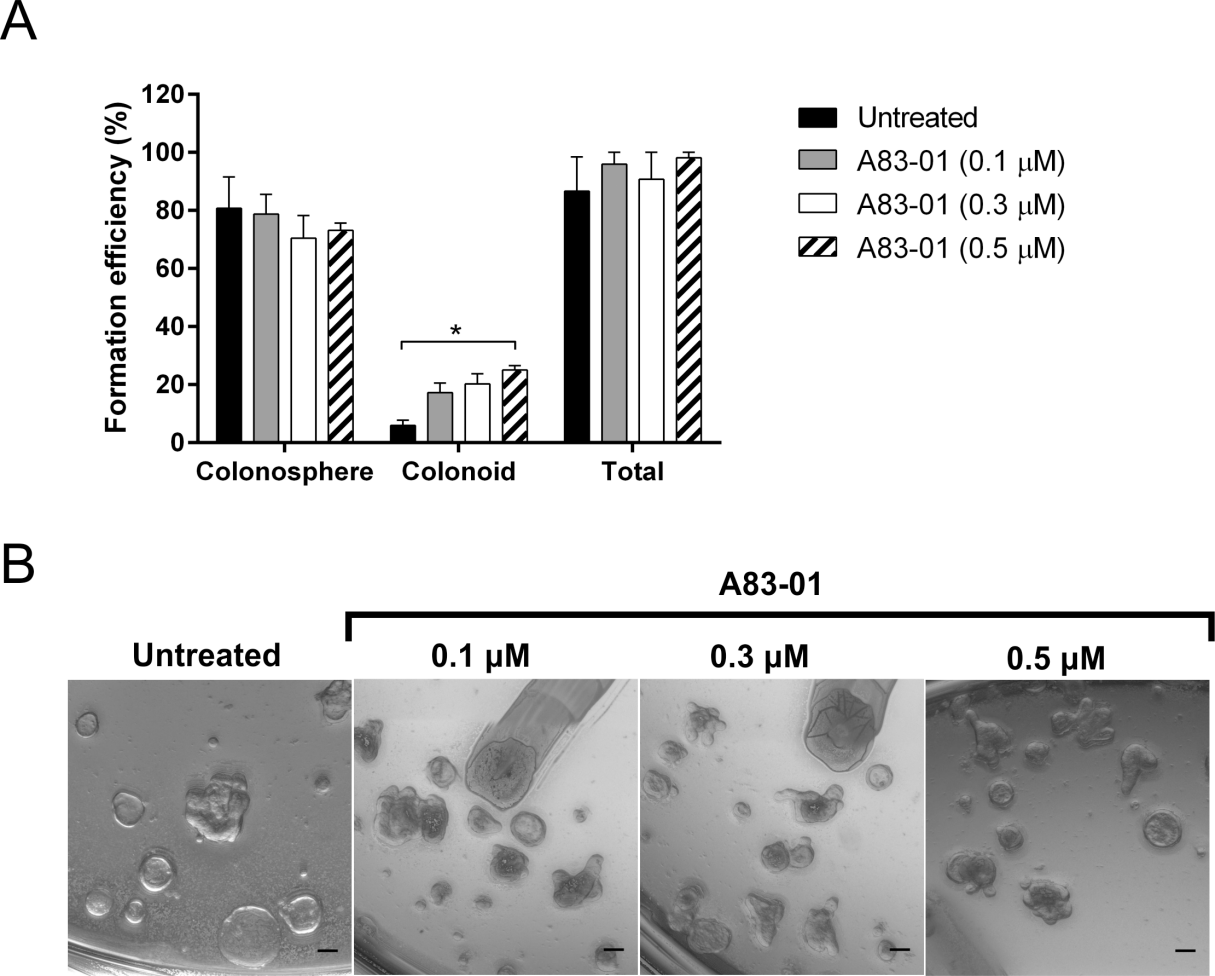
Inhibitors of TGF-β signaling enhanced colonoid formation. (A) A83-01, inhibitor of the TGFβ receptor kinase, was assayed at 0.1, 0.3 and 0.5 μM in duplicate culture of colon crypts with cYH2CM (50% v/v). The numbers of colonospheres and colonoids were counted on Day 6 and the colonosphere and colonoid formation efficiencies were determined. (B) Representative images of cultures on day 6. Error bars and analysis: Mean ± SEM, n = 3, * p<0.05, scale bar = 100 μm. 2-way ANOVA Fisher’s LSD test.

The TGFβ/Smad signaling is known to play a role in the proliferation and differentiation of intestinal epithelial cells [42]. Since it is likely that TGFβ would inhibit colonosphere formation, cYH2CM would be more likely to contain an antagonist of TGFβ action. We used A83-01 [46] (a TGF-β RI kinase inhibitor) to inhibit TGFβ signaling: A83-01 was added in 50% cYH2CM on day 0 and every other day thereafter. The cultures were imaged and formation efficiencies of colonospheres and colonoids were scored on day 6 (**Fig 8**). There was no significant effect of A83-01 on colonosphere formation, although the inhibitor did increase the proportion of colonoids (**Fig 8**). Thus, YH2CM does not appear to contain TGFβ like proteins (inhibition does not affect the efficiency of colonosphere formation), but some of its crypt stimulating activity might be associated with inhibition of TGFβ signaling (increased organoid with crypts formation).

EGF increases the proliferation of mouse [43] and Drosophila [47] intestinal stem cells. Consequently, EGF is included in the culture medium of small intestinal [23] and colon organoid cultures [24]. The budding efficiency of enteroids in culture correlates with the amount of EGF added [48].

**Fig 9:**
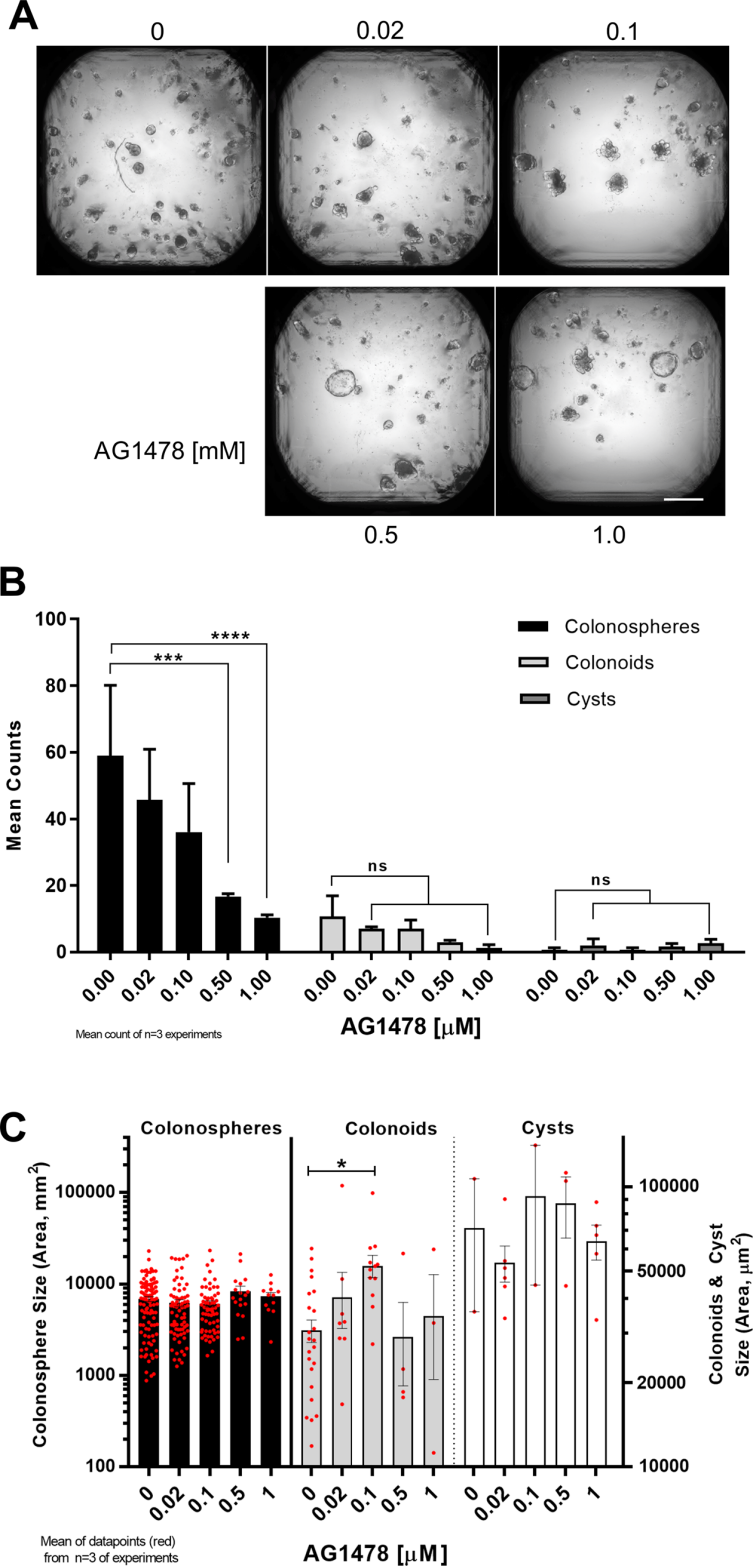
Optimum EGFR signaling is required for colonosphere viability and colonoid formation. The tyrphostin AG1478 was titrated with 30% v/v cYH2CM without EGF into colon crypt cultures grown in the wells of a 384 well plate, and were imaged on Day 5, quantitated and tabulated. (A) Representative bright field *Extended Depth of Field* (EDF) images of the colon crypt cultures showing the decreasing counts with increasing AG1478. The (B) number and (C) size of colonospheres, colonoids and cysts (a spheroid with a large and transparent pseudolumen). (B) Colonosphere counts decrease with increasing AG1478 concentration. (C) An addition of 0.1μM AG1478 leads to an increase in colonoid size. Scale/Error bars and analysis: 500μm. Mean ± SEM, n = 3 experiments, 2-way ANOVA, Dunnett's multiple comparisons test.

In the absence of EGF, cYH2CM or WEHI-YH2 cell feeder layers, even in the presence of Wnt and R-spondin, colon crypts do not form colonospheres or colonoids (S5A and S5B Fig). To investigate the role of EGFR-mediated signaling in the colon crypt cultures stimulated by cYH2CM, the EGFR tyrosine kinase inhibitor AG1478 [49] was added to the colon crypt cultures in the absence of EGF but together with cYH2CM (30%, v/v). AG1478 inhibits the EGFR with an EC_50_ of 80nM [49]. After adding the inhibitor and/or cYH2CM the colon cultures were followed for 5 days after which they were imaged, the colonospheres and colonoids were enumerated and the size distributions measured. In the absence of EGF, cYH2CM supports the formation of both colonospheres and organoids with crypts (**Fig 9A–9C**). Increasing the inhibition of the EGFR with increasing concentrations of AG1478 reduces the formation of colonospheres significantly, with no significant differences in the number of colonoids (**Fig 9A and 9B**). At 100nM AG1478 there was a significant increase in the average size of the colonoids compared to without the inhibitor. Large cyst formation occurred sporadically across the various concentrations of AG1478 (**Fig 9C**). cYH2CM appears to contain factors which can replace the requirements for the stimulation of the EGFR (S5C and S5D Fig). There appears to be an optimal level of EGFR stimulation for producing large colonoids (**Fig 9C**).

## Discussion

The formation and maintenance of crypts in the normal intestinal mucosa requires several biological processes: stem cell proliferation, self-renewal and differentiation; transit zone proliferation and differentiation; new crypt formation (budding)[9] and wound healing responses [6]. The production of cells within a crypt often occurs from LGR5+ stem cells under the stimulation of R-spondin/LGR5, Wnt/Frizzled, delta/notch and EGF/EGFR systems [18], however the regulatory systems controlling **new** crypt production and/or wound healing responses have not been identified in normal, adenomatous or cancers intestinal mucosa. In the normal mucosa < 0.5% of crypts are budding [9,50], yet all the crypts are producing large numbers of cells. Since the identification of a Matrigel culture system [23] which facilitates the spontaneous initiation and maintenance of crypt budding from small intestinal stem cells, it has become possible to investigate the factors which influence the initiation of both crypt production and the kinetics of cell production within a crypt. Whilst colon crypts form spheres in the intestinal Matrigel culture system [24], the production of new crypt buds in colon organoids is much less efficient than in the small intestinal organoid cultures [39].

We have reported previously that WEHI-YH2 cells used as feeder-layers stimulate the formation of both colonospheres and organoids from murine colon crypts [29]. Although the conditioned medium from WEHI-YH2 cells (YH2CM) contains proteins/factors that stimulate crypt formation *in vitro* (CoSFs), the low levels of the CoSFs in the WEHI-YH2 CM requires that the medium be concentrated to detect the activity. Previously, E16.5 embryonic intestinal myofibroblasts have been used as feeder layers to stimulate colon crypt formation [30]; Whilst the embryonic feeder layers supported the production of crypts after 12 days, WEHI-YH2 feeder cells (or cYH2CM) stimulate new colon crypts to form within 6 days. Intestinal crypt culture systems produce colonospheres, but the proportion of organoids with crypt buds has not been reported [24,34,51–53]. Although Sato and colleagues [24] described organoid forming efficiency from distal colon crypts as 50% and Ahmad *et al* [31] reported the formation of colon organoids with crypts at an efficiency of 33±5%), it is not clear from these reports whether the authors are referring to colonospheres or organoids with crypts. Such reports typically do not distinguish between colonospheres and organoid structures where crypts have formed in their calculations of organoid forming efficiency. In our experience, the proportion of mouse organoids which bud in the absence of mesenchymal cells (or YH2CM) is less than 1% of the crypts plated. In the presence of concentrated YH2CM, the proportion of colon organoids which form crypt buds increases to ~13%.

This report shows that the WEHI-YH2 feeder layers can be replaced by cYH2CM [29,30]. The WEHI-YH2 co-culture system requires the preparation of a confluent feeder layer before adding the Matrigel and colon crypts. However, with the feeder layer present, the Matrigel tends to lift off from the wells after a week, possibly due to the contractile movement or secretions from the WEHI-YH2 cells [54]. The WEHI-YH2 cells may also be detrimental to the growth of colonoids as they compete with the colon epithelial cells for nutrients. Our current culture system is the first colon organoid system to use myofibroblast conditioned medium to stimulate the production of new crypts in culture with high efficiency. When concentrated conditioned medium (cYH2CM) is used, the colonospheres and organoids with crypt buds are larger and more multi-lobular than the colonospheres grown from crypts in the absence of the cYH2CM. Indeed, the organoids grown in the presence of cYH2CM are usually larger than the organoids grown using feeder-layers of WEHI-YH2 cells.

Intestinal myofibroblasts are known to express many growth factors and cytokines (reviewed in [55,56]). We found that the CoSFs derived from WEHI-YH2 myofibroblasts are likely to be proteins. However, it is clear that cYH2CM contains several growth factors and that different growth factors may be responsible for cell production and the initiation of crypt budding. We have eliminated Wnt, R-spondin, BMP and TGF-β as the stimulators in cYH2CM responsible for the increased crypt budding.

The intestinal mesenchyme appears to express a series of Wnts [57], which may rescue the small intestinal epithelial stem cells from Paneth cell depletion. However, the cYH2CM neither contains Wnt3a nor does it activate canonical Wnt signaling. *de novo* crypt formation in the organoid cultures is possibly associated with the activation of quiescent stem cell/progenitor cells (e.g. Lrig1 or Dclk1 stem cells [48]) factors in the cYH2CM.

Activation of TGFβ/Smad signaling pathway can induce apoptosis in intestinal stem cells [58] and cell cycle arrest [59]. Deletion of Smad3, a TGFβ signaling transducer, leads to increased proliferation in the colon crypt progenitor cells [60]. Similarly, activation of BMP signaling pathway leads to apoptosis of colon epithelial cells [61] and inhibition of stem cell self-renewal through the suppression of Wnt/β-catenin signaling pathway [41]. Although BMPs have been detected in human colon crypts [44], BMP antagonists such as gremlin 1, gremlin 2 and chordin-like 1 are expressed in the surrounding human colon myofibroblasts of the colon epithelial stem/progenitor cells at the bottom of crypts [44]. These observations formed the basis of adding a BMP antagonist, noggin, to the crypt cultures [23,24] to prolong the *in vitro* culture by maintaining the stem cells. The expression of BMP signaling transducers (phospho-Smad 1,5 and 8) and TGFβ signaling transducers (phospho-Smad 2 and 3) are suppressed at the bottom of human colon crypts [45], suggesting that both Bmp and TGFβ signaling pathway are suppressed at the bottom of crypts. In agreement with expression studies in human colon crypt cultures by Reynolds and colleagues [45], our *in vitro* experiments showed differential increase in colonoids using the TGFβ receptor kinase inhibitor A83-01 (i.e. rescued TGFβ-induced suppression of stem/progenitor cell proliferation) and decreased colonoids with BMP4 (i.e. BMP4 induced suppression).

EGF signaling pathway is activated in the intestinal epithelial stem cell niche [62]. EGF enhances cell proliferation of a mouse adult intestinal progenitor cell line [43], intestinal stem cells in Drosophila [47] and the mouse embryonic intestine [63]. Inhibition of the EGF signaling pathway by using AG1478 mesylate in colonoid cultures shows there is an optimal level of EGF signaling for crypt bud formation but the inhibition of the EGFR did not affect the size of colonospheres, suggesting that after colonosphere initiation cell proliferation was not compromised in the presence of the EGFR kinase inhibitor. Detailed analysis of organoid formation in the cultures reveals distinct morphological phases: early colonosphere initiation and the growth of the colonospheres to oblate ellipsoid structures. Depending on the concentration of EGF-like ligands, the colonospheres continue growing into either larger ellipsoids, initiate crypt bud formation or form large cysts. cYH2CM stimulates colonoid formation *in vitro*, providing a critical platform for studying the effects of the EGF, BMP, Notch, TGFβ and inflammatory cytokine signaling pathways on colon crypt formation (i.e. organoids). The efficient formation of normal colon crypt buds *in vitro* depends on the presence of Wnt, R-spondin, an optimal concentration of a ligand capable of stimulating the EGFR and CoSFs secreted from myofibroblasts. It will be interesting to compare crypt bud production from normal, adenomatous and cancerous colon stem cells in the presence of the CoSFs and to identify the growth factors in cYH2CM responsible for stimulating colon crypt formation *in vitro*.

## Materials and methods

### Animals

The C57BL/6 mice were bred under specific pathogen-free conditions and maintained by Walter and Eliza Institute Bioservices. CO_2_ was used to anesthetize the mice and sacrifice was performed. All animal experimental procedures were approved by the Walter and Eliza Hall Institute Animal Ethics Committee (Ethics approval no. 002/11). Small intestinal and colon crypts were isolated from male mice at 6 and 7 weeks of age.

### Isolation of murine colon and small intestinal crypts

Detailed procedures to isolate normal murine colon crypts have been described previously [29]. In brief, the colons were sterilized in 0.04% (w/v) sodium hypochlorite solution for 10 minutes at room temperature, followed by crypt isolation buffer (2mM EDTA/0.5 mM DTT in PBS) in a 50ml tube for 40 minutes in a 37°C water bath. The colons were transferred to PBS and the crypts were released into the PBS by manual shaking and pelleted at 100 x g for 1 minute at 4°C. Similarly, the small intestines were incubated in crypt isolation buffer for 5 minutes at 37°C. The small intestines were transferred into PBS and the villi were released by manual shaking 20 times (discarded). The small intestinal fragments were then transferred and incubated in fresh crypt isolation buffer for another 20 minutes at room temperature. The small intestines were again transferred into PBS and crypts were released by manual shaking 20 times. This was repeated for 3 times, with the first fraction discarded and the second and the third fractions pooled and pelleted at 100 x g for 1 minute at 4°C.

### Classification of organoid cultures

The different organoid structures which appear in the cultures from isolated colon and small intestinal crypts are defined as follows [27]: **Colonosphere:** a spherical structure that composed of several types of colonic-epithelial cells (S1A Fig). It can be formed from either isolated single cells or crypts from the colon. **Colonoid:** a multilobulated structure with a pseudolumen that develops from colonosphere by forming one or more buds (S1B Fig). **Enterosphere:** a spherical structure that composed of several types of small intestinal epithelial cells (S1C Fig). It can be formed from either isolated single cells or from small intestinal crypts. **Enteroid:** a multilobulated structure with a pseudolumen that develops from an enterosphere by forming one or more buds (S1D Fig). **Cyst:** a large enterosphere with a transparent pseudolumen and a thin lining of epithelial cells as observed under a bright-field microscope (S1E Fig).

### Murine colonoid and enteroid cultures

For the co-cultures of WEHI-YH2 cells/colon crypts, WEHI-YH2 cells (4000 cells/well) were seeded in complete DMEM (Life Technologies Corporation #11995073) in 96-well plates and incubated overnight. The medium was aspirated and the cells were washed once with pre-warm Growth Medium-I [1X N2 supplement (Life Technologies Corporation #17502048) and 1X B27 supplement (Life Technologies Corporation #17504) in DMEM/F12 medium (Life Technologies Corporation #10565042) supplemented with penicillin-streptomycin solution (1% v/v)]. The crypt suspension (100 crypts/15 μl Matrigel) was added to each well on the top of the monolayer of WEHI-YH2 cells. The Matrigel (BD Biosciences #356231) was allowed to polymerize at 37°C for 30 minutes. 100 μl Growth Medium-II [Growth Medium-I with 50 ng/ml recombinant mouse EGF (Peprotech #315–09), 100 ng/ml recombinant human noggin (Peprotech #120–10), 10 μM Y-27632 (Sigma-Aldrich #Y0503), human R-spondin 2-Fc conditioned medium (1% v/v) and Wnt3a conditioned medium (50% v/v) or partially purified Wnt3a conditioned medium (1% v/v)] was added to each well. Fresh Growth Medium-II was replaced every two days. After day 4, Y-27632 was omitted from the cocktail. For colonoid cultures using WEHI-YH2 conditioned medium, 2X Growth Medium-II was prepared and mixed with 0 to 50% (v/v) of WEHI-YH2 conditioned medium. Recombinant human Bone Morphogenetic Protein 4 (BMP4) (Gibco #PHC9534), TGF-β RI kinase inhibitor VI A83-01 (Tocris #2939) or EGFR tyrosine kinase inhibitor Tyrphostin AG1478 mesylate (Institute of Drug Technology (IDT) Australia, Batch DA68001) were added to the cultures as indicated in the individual experiments. Enteroid cultures were maintained under the same conditions as colonoid cultures except Wnt3a was not added. For some experiments (i.e. EGF perturbation and enteroid cultures) where the cultures were grown in 384-well plates (Corning™ #3707), ~40 crypts were embedded in 6 μl Matrigel were seeded in 384-well plates and 40 μl of Growth Medium II was added and changed every 2 days.

R-spondin-Fc Conditioned Medium was harvested from 293F cells (after 7 days) transiently transfected with a human R-spondin2 construct that has Fc-fusion protein fused to the C-terminus and cloned into pApex vector.

### Image acquisition, processing and scoring

The detailed image acquisition and analysis procedures for acquiring images in 96 well plates have been described previously [25,29]. Briefly, bright field microscopy was conducted using a Nikon Eclipse Ti-U microscope with a 4x objective lens and images were captured on a Photometrics CoolSnap HQ CCD camera (Roper Scientific) using the ImageJ/Fiji software with the micro-manager software (version 1.3). Images of 12 fields of view per well at different planes of focus were stitched and stacked using the extended depth of focus (EDF) algorithm in ImageJ/Fiji software [64]. For the EGF perturbation and enteroid culture experiments, 384 well plates (Corning™ #3707) were used. The microscopy was conducted using a Nikon Eclipse Ti-U microscope with a 4x objective lens and motorised stage (Prior Scientific, H117). Bright field image stacks of each well were captured on a Nikon DS-Ri2 camera (Nikon Inc, MQA17000) using the NIS-Elements software (Nikon, Basic Research version 4.40). The depth images of each FOV (well) were then stacked using the EDF algorithm in Fiji. The processed images were analyzed manually and the objects identified were labelled as S (colonospheres and enterospheres), O (colonoids and enteroids) or C (Cyst). The parameters were measured by drawing a line around the object. The colonosphere/enterosphere-forming efficiency was calculated as the percentage of the number of colonospheres/enterospheres (S) formed from the number of crypts plated at day 0. Likewise, the colonoid/enteroid-forming efficiency and cyst-forming efficiency was calculated as the percentage of the crypts plated at day 0.

### Preparation and concentration of WEHI-YH2 conditioned medium (YH2CM)

WEHI-YH2 cells were seeded in tissue culture flasks or dishes and incubated in DMEM supplemented with 10% heat inactivated fetal bovine serum (HI-FBS) until the cultures were 90% confluent. The medium was aspirated and fresh Growth Medium-I or plain DMEM/F12 medium were added to the cultures. The cultures were incubated for 24 to 72 hours depending on individual experiments. YH2CM was harvested and spun at 1300 x g for 5 min before filtering through a 0.2 μm filter for sterilization. The YH2CM was concentrated either by Centriprep centrifugal devices (Millipore) or a Vivaflow 50R ultrafiltration system (Sartorius Stedim Australia) according to the manufacturer’s instructions. The molecular weight cut-off for the membrane depended on the individual experiments.

### Inactivation of YH2CM

For thermal denaturation, the YH2CM was heated at 68°C for an hour in a dry block heater. For trypsin digestion, a trypsin stock (25 mg/ml) was prepared fresh by dissolving lyophilized trypsin (Life Technologies #27250–018) in PBS and added to YH2CM at 50 μg/ml working concentration. For the negative control, the trypsin stock was inactivated by heating at 95°C for 10 min. The reaction mixtures were incubated at 37°C for 3 hours. A stock of 10 mg/ml trypsin inhibitor (Sigma-Aldrich #T6522) was added to the reaction mixtures to a final concentration of 400 μg/ml in order to stop the activity of trypsin. The reaction mixtures were sterilized using 0.2 μm filters and applied to the respective experiments.

### Western blot analyses

To assay for the production of NICD (i.e. Notch activation), the HCT116 cell line was plated in 6-well plate and treated with YH2CM or 10 mM EDTA. Cells were washed twice in ice-cold PBS and lysed in Lysis Buffer (25 mM Tris·HCl pH7.5, 150 mM NaCl, 1% NP-40, 1% Sodium deoxycholate, 0.1% SDS and 1X protease inhibitor cocktail (Roche Applied Science #04693159001)) for 10 min on ice. The cells were scraped off the wells and transferred to 1.5 ml tubes. The cell lysate was clarified at 13,000 rpm for 60 min at 4°C. The supernatants (cell lysates) were transferred to new 1.5 ml tubes. The protein concentration of each sample was determined using Pierce BCA protein assay (Thermo Fisher Scientific #23227). The same amount of total proteins was loaded in each lane. The samples were mixed with NuPAGE® LDS Sample Buffer (4X) and 0.5 M dithiothreitol (10X) and boiled at 95°C for 5 min.

For the β-Catenin stabilization assay (i.e. to detect Wnt activity), L-cells were cultured in 6-well plate and treated with partially purified Wnt3a CM or concentrated YH2CM for 4 hours. Cells were lysed directly in NuPAGE® LDS Sample Buffer (4X) (Life Technologies #NP0007) and 0.5 M dithiothreitol (10X). Protein were separated in NuPAGE® Novex® Bis-Tris 4–12% gel (Life Technologies #NP0322BOX) by SDS-polyacrylamide gel electrophoresis, followed by transferring the proteins onto a PVDF membrane (Bio-rad #1620177) at 90 V for 3 hours at 4°C. The PVDF membranes were blocked in 5% (w/v) skim milk in TBS and the proteins of interest were detected using the following primary antibodies: mouse anti-human/mouse β-catenin monoclonal antibody (Clone 14/ β-catenin, BD Transduction Laboratories #610153), rabbit anti-mouse cleaved Notch 1 monoclonal antibody (Clone D3B8, Cell Signaling #4147) and the mouse anti-human/mouse β-tubulin monoclonal antibody (Clone 5H1, BD Pharmingen #556321); and secondary antibodies: IRDye® 800CW goat anti-rabbit polyclonal IgG (H+L) and IRDye® 800CW goat anti-mouse polyclonal IgG (H+L) (LI-COR Biosciences #926–32211 and 926–32210). LICOR Odyssey Infrared Imaging System (LI-COR Biosciences) was used to image the membrane according to the manufacturer’s instructions.

### FACS-sorted colon crypt cells co-culture

Isolated colonic crypts were dissociated into single cells by incubating in DMEM/F12 media containing 0.3 U/ml dispase and 2000 U/ml DNase I at 37°C for 20 minutes. Cells were then dispersed in a 21-G needle for further dissociation on ice. Dispase reaction was stopped by adding excess PBS. Cell clumps were removed by a 40-μm cell strainer. The cell suspension was pelleted at 1300 x g for 5 minutes at 4°C and resuspended in FACS buffer (2 mM EDTA, 1% (v/v) FBS and 10 U/ml DNase I). The cells were then stained with Sytox Green viability dye (1:1500; #S34860 ThermoFisher), CD31-FITC (1:100; #102405 Biolegend) and CD45-FITC (1:100; #553080 BD Pharmingen) antibodies to exclude dead cells, endothelial cells and hematopoietic cells, respectively. EpCAM-PE/Cy7 (1:200; #118215 Biolegend), CD44 (1:200; #103008 Biolegend) and CD24 (1:800; #101814 Biolegend) antibodies were used to purify colonic cells into different subsets using a BD FACSAria II with a 100 μm nozzle. 4000 of the sorted cells per well were embedded in 15 μl Matrigel and co-cultured on the top of a monolayer of WEHI-YH2 cells in a 96 well plate.

## Supporting information

S1 Fig Images of murine colon and small intestinal organoids. Representative images of the different distinctive structures observed in the colon and small intestinal organoid cultures. The top panels show (A) a colonosphere and (B) a colonoid on Day 6 in colon crypt culture. The bottom panels show (C) an enterospheres, (D) an enteroid and (E) a cyst on Day 4 of a small intestinal crypt culture. Scale bar = 100 μm.
(TIF)

S2 Fig The ability of concentrated YH2CM to stimulate colon crypt formation (*colonoid*) is independent of the supplements N2 or B27. Conditioned medium from WEHI-YH2 cells (YH2CM) was collected from confluent cultures which had been incubated for 48 hours in the respective medium with/without supplements. The collected conditioned medium (with/without N2 and B27) as well as DMEM with F12, N2 and B27 (DMEM-F12, N2, B27) were concentrated 10-fold (cYH2CM) using a Centriprep YM3 filter (with a 3kDa molecular weight cut-off). These concentrates were tested for their ability to grow colon crypt cultures (50% v/v) in comparison with WEHI-YH2 colon crypt co-culture. Images of the cultures were taken, processed and the colonoid and colonosphere forming efficiencies (Day 6 compared to Day 0) were quantitated. Error bars and analysis: n = 3 for DMEM-F12, N2, B27 and cYH2CM-F12, N2, B27 (paired t-test, two-tailed p-value = 0.0107).
(TIF)

S3 Fig Quantitation of colonoid average counts and average buds per colonoid. (Supplementary Figure for Fig 3) Representative *Extended Depth of Field* (EDF) images of day 6 colon cultures with 12.5% cYH2CM. The number of crypt buds per colonoid was scored and labelled at the bottom-right corner of each image. A total of 45 crypts from 21 colonoids were counted from duplicate sets of cultures in three independent experiments. Therefore, Colonoid Average Counts: 21÷6 = 3.5; Average Buds per colonoid: [(29÷12) + (6÷1) + (10÷8)] ÷3 = 3, where 12, 1 and 8 are the total number of colonoid for each of the three independent experiments. The arrows indicate the position of each crypt bud of the colonoid. The scale bar = 100 μm.
(TIF)

S4 Fig WEHI-YH2 cells do not express or secrete Wnt3a or R-spondin-2. WEHI-YH2 cell lysate (45 μg), concentrated YH2CM (cYH2CM) (7.5 μg), R-spondin 2-Fc CM (5 μl) (see Materials and Methods), recombinant human (rh) R-spondin 2 (1 μg) (R&D systems, #3266) and partially purified Wnt3a CM (pWnt3a) (1 μl) were loaded into different lanes of a 4–12% Bis-Tris gel and run using SDS-PAGE. The protein expression of R-spondin 2 was detected using an anti-R-spondin 2 antibody (R&D systems, #AF3266) and a donkey anti-goat IgG (H+L)-Alexa Fluor 647 conjugate secondary antibody (Life Technologies, #A-21447); the expression of Wnt3a was detected using an anti-Wnt3a antibody (Cell Signaling Technology, #2391S) and an IRDye® 800CW goat (polyclonal) anti-mouse IgG (H+L) secondary antibody (LI-COR, #926–32210) in immunoblots. The two protein bands detected in the lane of R-spondin 2-Fc CM indicates that the R-spondin 2-Fc protein is partially cleaved. M: protein standards.
(TIF)

S5 Fig WEHI-YH2 contains factors which substitutes for EGF stimulation required in colon crypt culture. Colon crypts were cultured (A-B) without WEHI-YH2 feeder cells/YH2CM or (C-D) with WEHI-YH2 feeder cells. Control media (A and C) include all growth factors and inhibitors (including EGF) while B and D contains no EGF. Without WEHI-YH2 (feeder or CM), cultures without EGF do not survive, indicating the requirement of colon culture for EGF. On the other hand, co-culture with WEHI-YH2 appears to substitute for the requirement of exogenous EGF. Representative images shown (n = 3) from day 6 cultures.
(TIF)

S6 Fig Effect of YH2CM on the budding frequency per organoid in small intestinal crypt cultures. Small intestinal crypts (60 crypts/wells) were cultured in different concentration (% v/v) of cYH2CM in triplicates in a 384 well plate for 4 days. (A) The number of enterospheres, enteroids and cysts and the number of buds of individual organoids were counted on day 4. The fraction (%) of each organoid type (categorized according to the number of buds and the morphology) was calculated. (B) More multilobular organoids and are formed when the concentration of cYH2CM increased. (C) The formation efficiency of total colonies and different types of organoids at different concentration of cYH2CM. Error bars and analysis: mean±SEM, n = 3, *p<0.05, ** p< 0.01, ***p<0.005, **** p<0.001 (treatment vs 0). A: 2-way ANOVA, Tukey's multiple comparisons test. C: 2-way ANOVA, Holm-Sidak’s multiple comparisons test. Scale bar: 500μm
(TIF)

S7 Fig YH2CM colon culture support and stimulation is species specific. Human colon crypts were grown in culture under the following conditions: (A) without any conditioned medium (control), (B) cYH2CM (30%, v/v), or (C) conditioned media from a human myofibroblast (30%, v/v). The A and C are negative and positive control respectively. Image stacks of the cultures were acquired on almost every day for 14 days with the respective representative images shown. The numbers of colonies were scored on Day 14 for four fields of views (FOV) and the average (D) size and (E) count of the colony were determined and tabulated. YH2CM provided limited support for human colon crypt growth *in vitro* (similar to control) as compared to that provided by the conditioned media from the human myofibroblast which has significant larger colonies. No significant difference in counts was observed between the different conditions, however YH2CM treatments do appears to have lesser colonies. This implies that the colonoid stimulating factors present in YH2CM are species specific. Error bars and analysis: Mean ± SEM, * p< 0.05, n = 4 FOV, scale bar = 100 μm, 1way ANOVA Dunnett's multiple comparisons test.
(TIF)

S1 Text Effects of cYH2CM on the formation of small intestinal crypts in vitro. (PDF)

S2 Text Actions of CoSF(s) from Murine WEHI-YH2 cells are species specific. (PDF)
