# Supplementary figures and images for: Colon organoid formation and cryptogenesis are stimulated by growth factors secreted from myofibroblasts
Source: PLoS One. 2018 Jun 21;13(6):e0199412. doi: 10.1371/journal.pone.0199412 (PMC6013242; doi:10.1371/journal.pone.0199412)

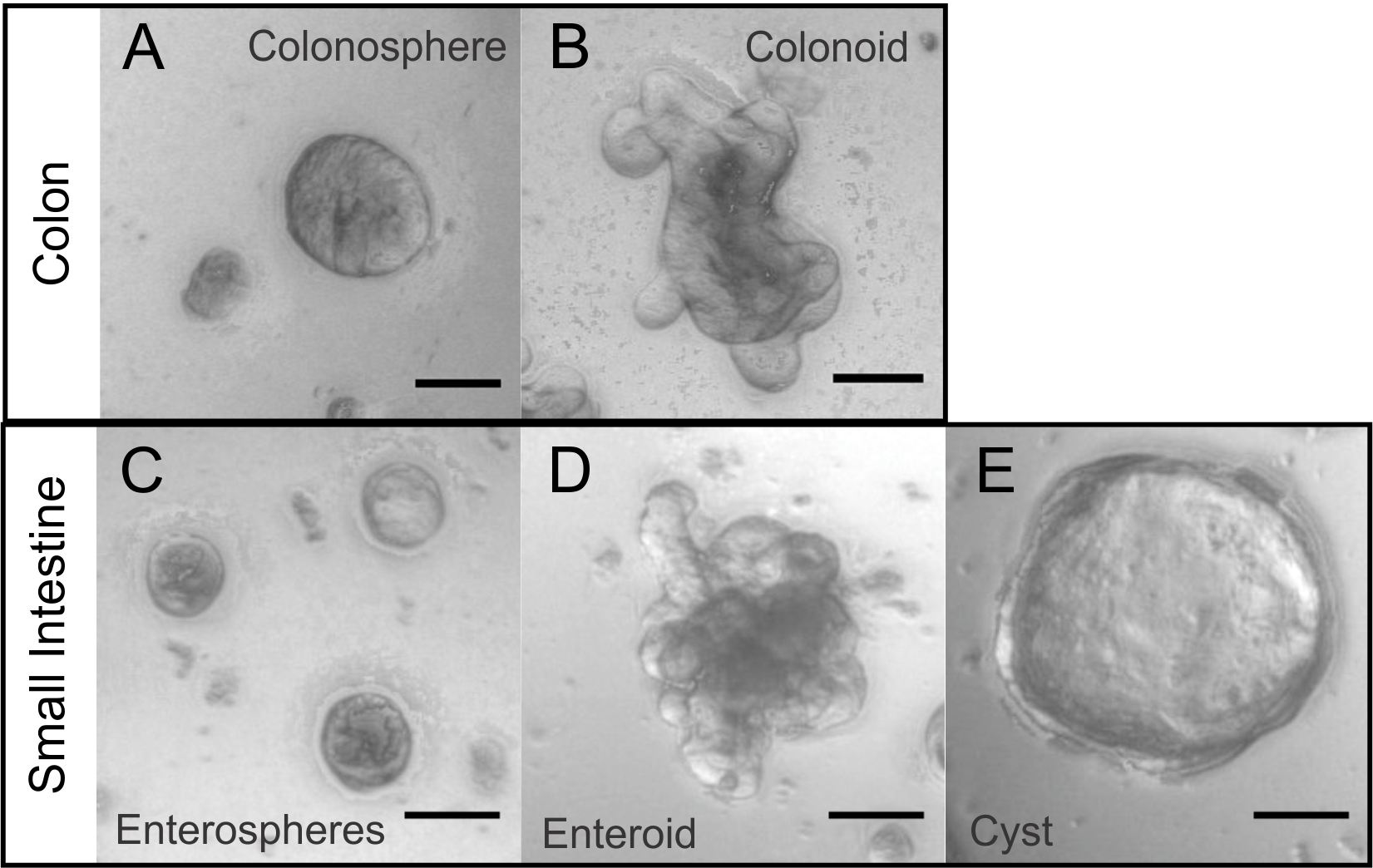

Supplement: S1 Fig — Representative images of the different distinctive structures observed in the colon and small intestinal organoid cultures. The top panels show (A) a colonosphere and (B) a colonoid on Day 6 in colon crypt culture. The bottom panels show (C) an enterospheres, (D) an enteroid and (E) a cyst on Day 4 of a small intestinal crypt culture. Scale bar = 100 μm. (TIF) [file pone.0199412.s001.tif]

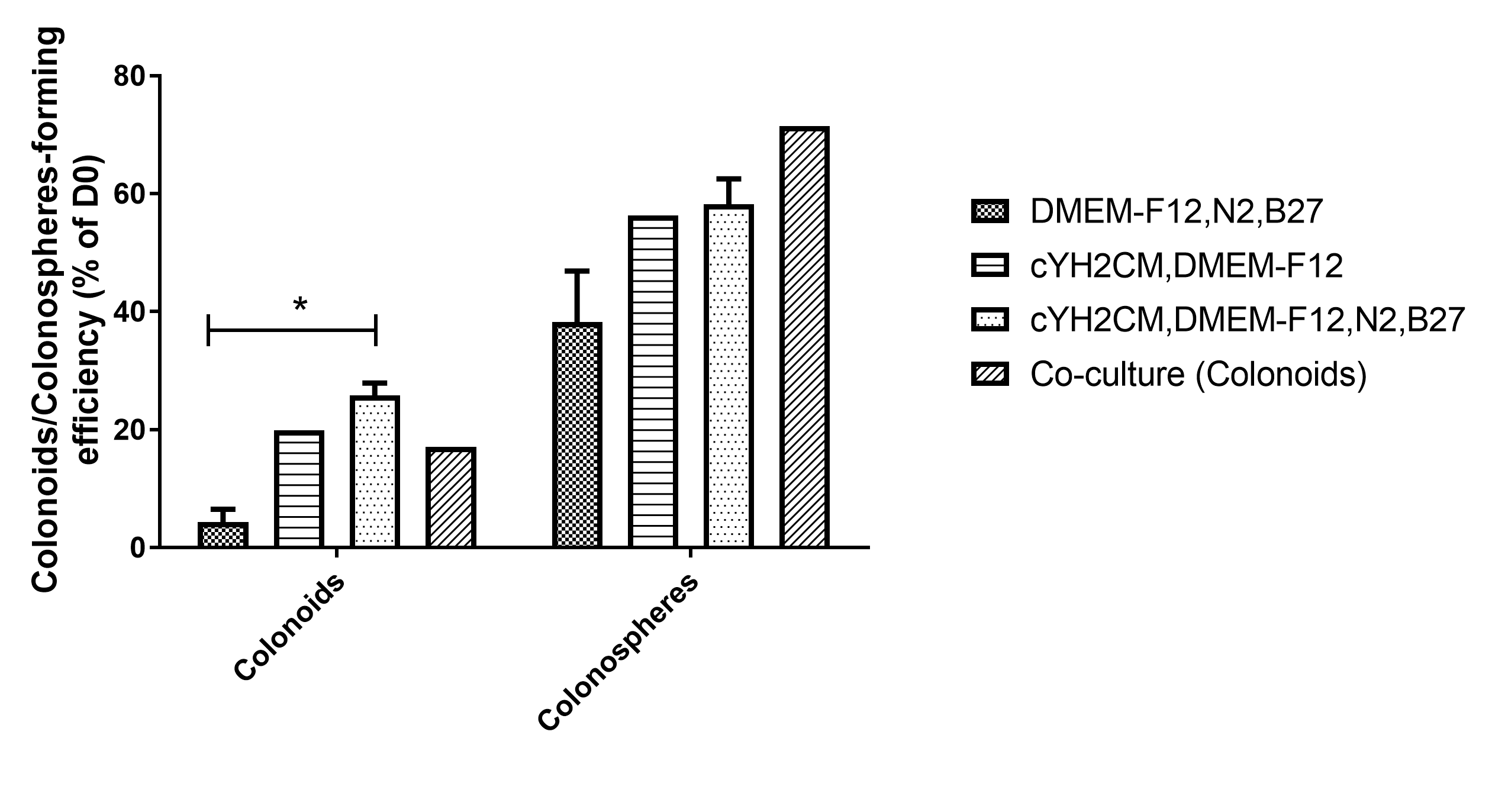

Supplement: S2 Fig — Conditioned medium from WEHI-YH2 cells (YH2CM) was collected from confluent cultures which had been incubated for 48 hours in the respective medium with/without supplements. The collected conditioned medium (with/without N2 and B27) as well as DMEM with F12, N2 and B27 (DMEM-F12, N2, B27) were concentrated 10-fold (cYH2CM) using a Centriprep YM3 filter (with a 3kDa molecular weight cut-off). These concentrates were tested for their ability to grow colon crypt cultures (50% v/v) in comparison with WEHI-YH2 colon crypt co-culture. Images of the cultures were taken, processed and the colonoid and colonosphere forming efficiencies (Day 6 compared to Day 0) were quantitated. Error bars and analysis: n = 3 for DMEM-F12, N2, B27 and cYH2CM-F12, N2, B27 (paired t-test, two-tailed p-value = 0.0107). (TIF) [file pone.0199412.s002.tif]

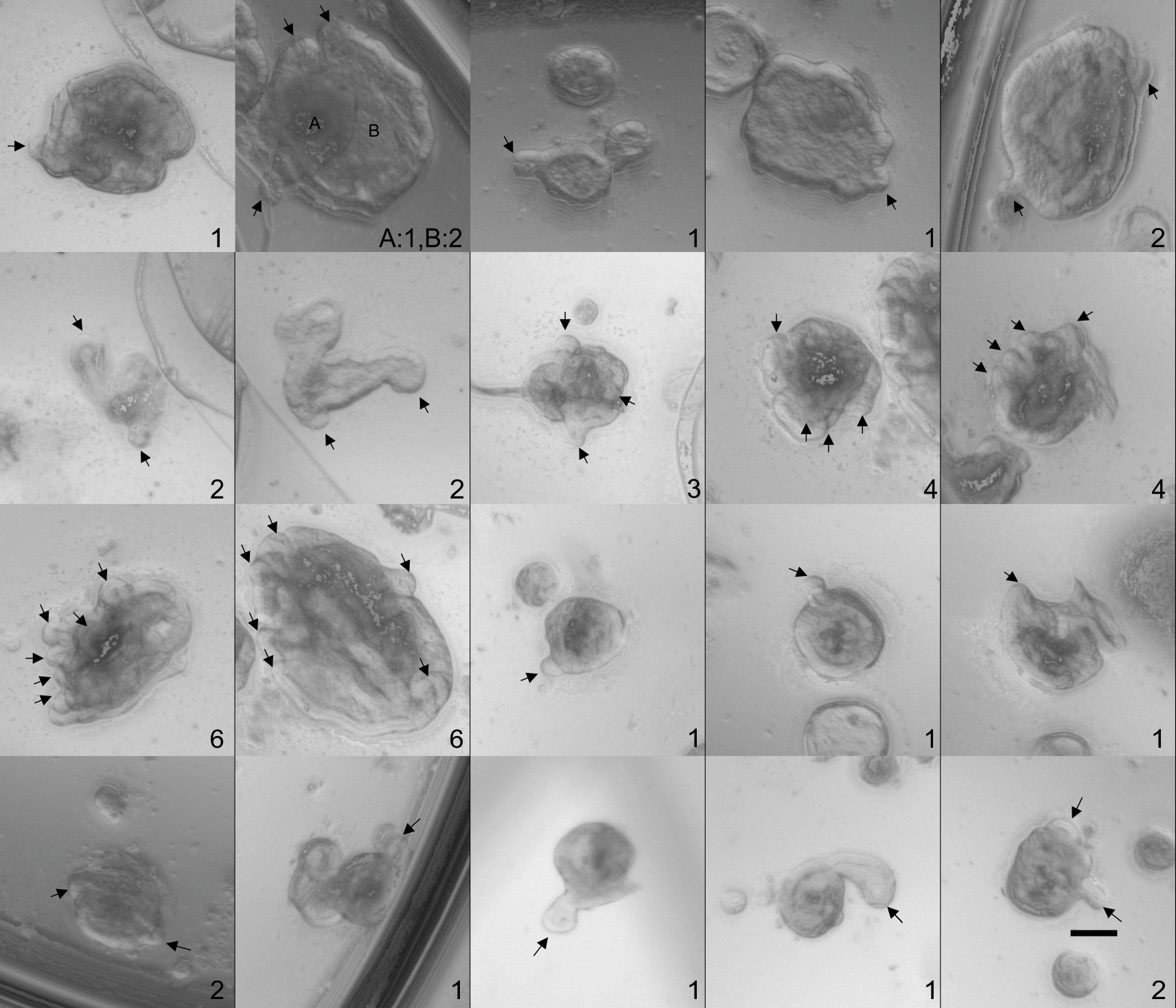

Supplement: S3 Fig — (Supplementary Figure for Fig 3) Representative Extended Depth of Field (EDF) images of day 6 colon cultures with 12.5% cYH2CM. The number of crypt buds per colonoid was scored and labelled at the bottom-right corner of each image. A total of 45 crypts from 21 colonoids were counted from duplicate sets of cultures in three independent experiments. Therefore, Colonoid Average Counts: 21÷6 = 3.5; Average Buds per colonoid: [(29÷12) + (6÷1) + (10÷8)] ÷3 = 3, where 12, 1 and 8 are the total number of colonoid for each of the three independent experiments. The arrows indicate the position of each crypt bud of the colonoid. The scale bar = 100 μm. (TIF) [file pone.0199412.s003.tif]

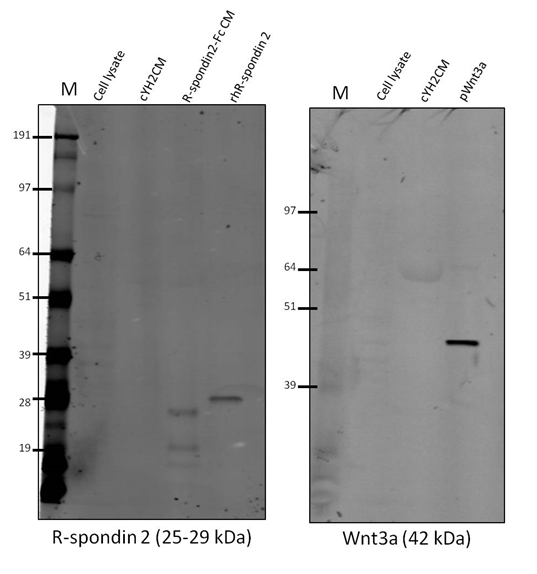

Supplement: S4 Fig — WEHI-YH2 cell lysate (45 μg), concentrated YH2CM (cYH2CM) (7.5 μg), R-spondin 2-Fc CM (5 μl) (see Materials and Methods), recombinant human (rh) R-spondin 2 (1 μg) (R&D systems, #3266) and partially purified Wnt3a CM (pWnt3a) (1 μl) were loaded into different lanes of a 4–12% Bis-Tris gel and run using SDS-PAGE. The protein expression of R-spondin 2 was detected using an anti-R-spondin 2 antibody (R&D systems, #AF3266) and a donkey anti-goat IgG (H+L)-Alexa Fluor 647 conjugate secondary antibody (Life Technologies, #A-21447); the expression of Wnt3a was detected using an anti-Wnt3a antibody (Cell Signaling Technology, #2391S) and an IRDye® 800CW goat (polyclonal) anti-mouse IgG (H+L) secondary antibody (LI-COR, #926–32210) in immunoblots. The two protein bands detected in the lane of R-spondin 2-Fc CM indicates that the R-spondin 2-Fc protein is partially cleaved. M: protein standards. (TIF) [file pone.0199412.s004.tif]

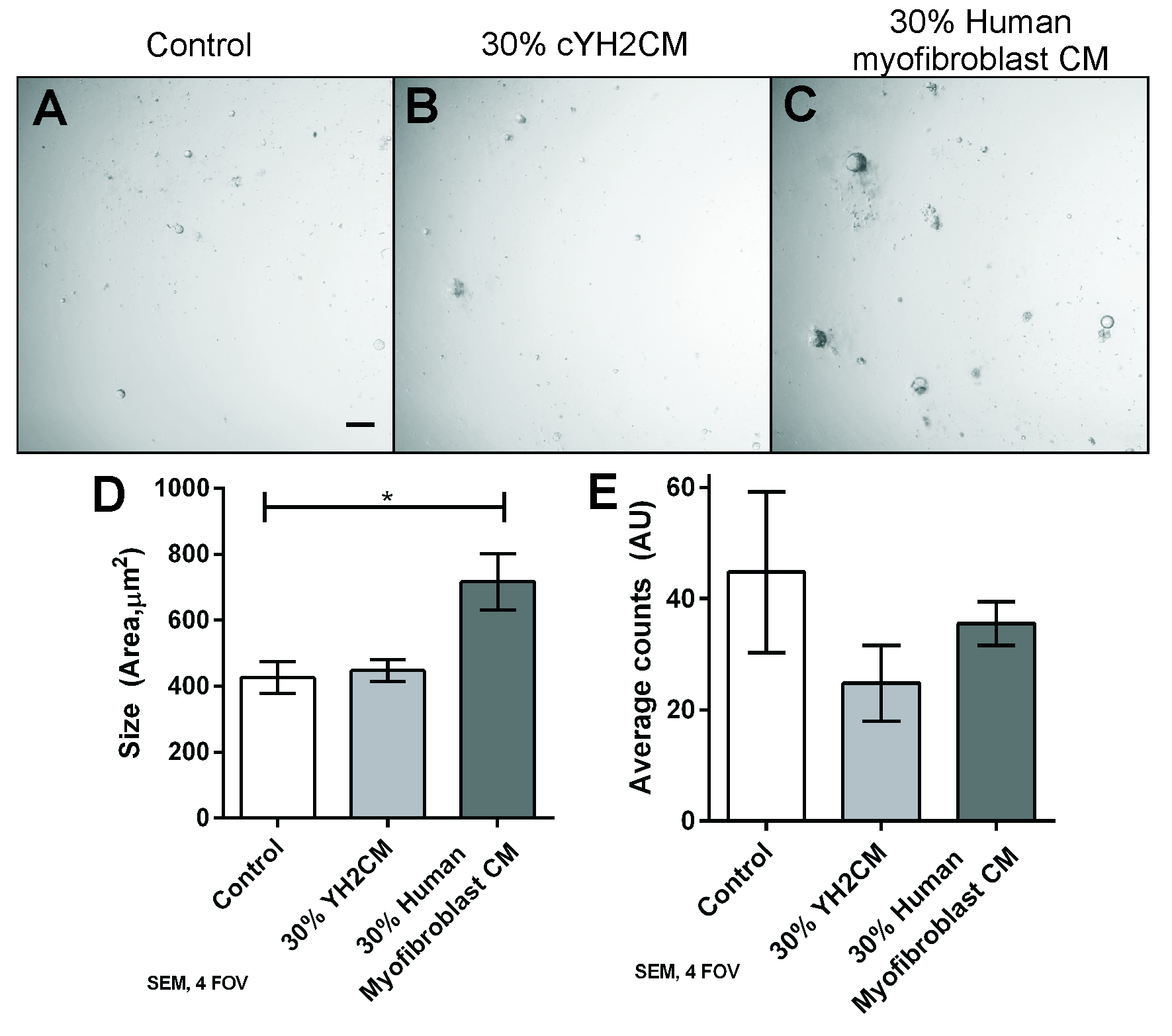

Supplement: S7 Fig — Human colon crypts were grown in culture under the following conditions: (A) without any conditioned medium (control), (B) cYH2CM (30%, v/v), or (C) conditioned media from a human myofibroblast (30%, v/v). The A and C are negative and positive control respectively. Image stacks of the cultures were acquired on almost every day for 14 days with the respective representative images shown. The numbers of colonies were scored on Day 14 for four fields of views (FOV) and the average (D) size and (E) count of the colony were determined and tabulated. YH2CM provided limited support for human colon crypt growth in vitro (similar to control) as compared to that provided by the conditioned media from the human myofibroblast which has significant larger colonies. No significant difference in counts was observed between the different conditions, however YH2CM treatments do appears to have lesser colonies. This implies that the colonoid stimulating factors present in YH2CM are species specific. Error bars and analysis: Mean ± SEM, * p< 0.05, n = 4 FOV, scale bar = 100 μm, 1way ANOVA Dunnett's multiple comparisons test. (TIF) [file pone.0199412.s007.tif]
