# Supplementary material for: Colon organoid formation and cryptogenesis are stimulated by growth factors secreted from myofibroblasts
Source: PLoS One. 2018 Jun 21;13(6):e0199412. doi: 10.1371/journal.pone.0199412 (PMC6013242; doi:10.1371/journal.pone.0199412)
Supplement: S1 Text — (PDF) [file pone.0199412.s008.pdf]

Supporting Information (S1 Text)

Effects of cYH2CM on the formation of small intestinal crypts *in vitro*

In normal culture condition without cYH2CM, *small intestinal* crypts form enterospheres (39±11%) as well as enteroids with less than or 3 buds (57±9%). Only a small percentage of enteroids have greater than 3 buds (5±2%) (S6A Fig, left most bar of each category)[23]. The cYH2CM, containing CoSFs, stimulates the size of both the enterospheres and enteroid with crypts (S6B Fig). Interestingly, increasing the cYH2CM concentration leads to more enteroids having greater than 3 buds (S6A and S6B Fig). At the highest cYH2CM concentrations (i.e. 50%, v/v), there appear to be more cysts (37±4% of the total counts) formed compared to enteroids having >3 buds (9±2%). At the highest cYH2CM concentration (50%, v/v) the increased proportion of cysts is associated with a lower proportion of enteroids with crypts. Increasing the cYH2CM concentration does not change the proportion of enterospheres (44±5%) (S6A Fig). The total organoid formation efficiency (enterosphere + enteroids + cysts) of the small intestinal crypt culture increases from 59±6% (with no cYH2CM) to 89±25% at 30% v/v of cYH2CM but then decreases to 54±7% at 50% v/v of YH2CM (S6C Fig).

Actions of CoSF(s) from Murine WEHI-YH2 cells are species specific

The CoSF(s) from the murine WEHI-YH2 cells are species specific. The cross-specificity of the colonoid-stimulating factors (CoSFs) in YH2CM was tested by culturing human colonoid cultures with cYH2CM (30%, v/v). cYH2CM did not stimulate the formation of human colon. In contrast, conditioned medium from a human

colon myofibroblast cell line (30%, v/v) stimulates the formation of human colon organoids (S7 Fig).
