# Supplementary material for: Colon organoid formation and cryptogenesis are stimulated by growth factors secreted from myofibroblasts
Source: PLoS One. 2018 Jun 21;13(6):e0199412. doi: 10.1371/journal.pone.0199412 (PMC6013242; doi:10.1371/journal.pone.0199412)
Supplement: S2 Text — (PDF) [file pone.0199412.s009.pdf]

Supporting Information (S2 Text)

Actions of CoSF(s) from Murine WEHI-YH2 cells are species specific

The CoSF(s) from the murine WEHI-YH2 cells are species specific. The cross-specificity of the colonoid-stimulating factors (CoSFs) in YH2CM was tested by culturing human colonoid cultures with cYH2CM (30%, v/v). cYH2CM did not stimulate the formation of human colon. In contrast, conditioned medium from a human colon myofibroblast cell line (30%, v/v) stimulates the formation of human colon organoids (S7 Fig).
